# Defending Against Advanced Persistent Threats Using Game-Theory

**DOI:** 10.1371/journal.pone.0168675

**Published:** 2017-01-03

**Authors:** Stefan Rass, Sandra König, Stefan Schauer

**Affiliations:** 1 Universität Klagenfurt, Institute of Applied Informatics, Klagenfurt, Austria; 2 Austrian Institute of Technology, Safety & Security Department, Klagenfurt, Austria; Tianjin University of Technology, CHINA

## Abstract

Advanced persistent threats (APT) combine a variety of different attack forms ranging from social engineering to technical exploits. The diversity and usual stealthiness of APT turns them into a central problem of contemporary practical system security, since information on attacks, the current system status or the attacker’s incentives is often vague, uncertain and in many cases even unavailable. Game theory is a natural approach to model the conflict between the attacker and the defender, and this work investigates a generalized class of matrix games as a risk mitigation tool for an advanced persistent threat (APT) defense. Unlike standard game and decision theory, our model is tailored to capture and handle the full uncertainty that is immanent to APTs, such as disagreement among qualitative expert risk assessments, unknown adversarial incentives and uncertainty about the current system state (in terms of how deeply the attacker may have penetrated into the system’s protective shells already). Practically, game-theoretic APT models can be derived straightforwardly from topological vulnerability analysis, together with risk assessments as they are done in common risk management standards like the ISO 31000 family. Theoretically, these models come with different properties than classical game theoretic models, whose technical solution presented in this work may be of independent interest.

## 1 Introduction

The increasing heterogeneity, connectivity and openness of today’s information systems often lets cyber-attackers find ways into a system on a considerably large lot of different paths. Today, security is commonly support by semi-automated tools and techniques to detect and mitigate vulnerabilities, for example using topological vulnerability analysis (TVA), but this progress is paired with the parallel evolution and improvements to the related attacks. APTs naturally respond to the increasing diversity of security precautions by mounting attacks in a stealthy and equally diverse fashion, so as to remain “under the radar” for as long as is required until the target system has been penetrated, infected and can be attacked as intended. Countermeasures may then come too late to be effective any more, since the damage has already been caused by the time when the attack is detected.

Mitigating APTs is in most cases not only a matter of technical precautions, but also some sort of fight against an invisible opponent and external influences on the system (coming from other connected systems but primarily due to the APT remaining hidden). Thus, any security measure taken may or may not be effective on the current system state, depending on how far the APT has evolved already. The question of economics then becomes particularly difficult and fuzzy, since the return on security investments is almost impossible to quantify in light of many factors that are outside the security officer’s scope of influence.

### 1.1 Related Work

In the last decade, the number of APTs [1] increased rapidly and numerous related security incidents were reported all over the world. One major reason therefore is that APTs are not focusing on a single vulnerability in a system (which could be detected and eliminated easily), but are using a chain of vulnerabilities in different systems to reach high-security areas within a company network. In this context, adversaries often exploit the fact that most of the protection efforts go into perimeter protection, so that moving inside the infrastructure is much easier and the attacker has a good chance to go unnoticed once being inside. Overcoming the perimeter protection by social engineering or malware (even unknowingly) carried inside by legitimate persons (bring-your-own-device problem) are only two ways to penetrate the perimeter security. Once the perimeter has been overcome, insider attacks are considered as an even bigger threat [2]. Extensive guidelines and recommendations exist to secure this internal area [3], e.g., the demilitarized zone (DMZ) but the intensity of the surveillance is limited. Specialized tools for intrusion detection or intrusion prevention require a large amount of administration and human resources to monitor the output of these systems.

APTs are characterized by a combination of several different attack methods (social engineering, technical hacks, malware, etc.) that is being tailored to and optimized for the specific organization, its IT network infrastructure and the existing security measures therein. Often, even yet not officially reported weaknesses, known as zero-day vulnerabilities, of the network infrastructure are in additional use. Especially the application of social engineering in the beginning stages of an APT lets the attacker bypass many technical measures like intrusion detection and prevention systems, so as to efficiently (and economically) get through the outer protection (perimeter) of the IT network. A prominent APT attack was the application of the Stuxnet malware in 2008 [4–6], which was introduced into Iran’s nuclear plants sabotaging the nuclear centrifuges. In the following years, other APT attacks, like Operation Aurora, Shady Rat, Red October or MiniDuke [1, 7, 8] have become public. Additionally, the Mandiant Report [9] explicitly states how APTs are used on a global scale for industrial espionage and that the attackers are often closely connected to governmental organizations.

The detection of APT attacks has therefore become an object of extensive research over the past years. As perimeter protection tools are occasionally failing to prevent intrusions, anomaly detection methods have been inspected to provide additional protection [10, 11]. The main idea is to detect the presence of an adversary inside an organization’s network, based on the adversary’s actions when it moves from one spot to another, or tries to access sensitive data (honeypots). Often, the detection rests on log file analysis, with data collected from all over the network and applications therein. Designated logging engines (e.g., syslog (http://tools.ietf.org/html/rfc5424) or logging management solutions (e.g., Graylog (http://graylog2.org/) are usually in charge here. Nevertheless, the detection of exceptional events in these log files alone is insufficient, since anomalies are often exposed not before events are correlated with each other [12, 13]. Since today’s systems are heavily connected and interchange a large amount of data on a regular basis, the size of the logging information increases drastically, making an evaluation quite difficult.

An example for a tool realizing this approach is AECID (Automatic Event Correlation for Incident Detection) [14, 15]. AECID enforces white-lists and monitors system events, their occurrences as well as the interdependencies between different systems. In the course of this, the system is able to get an overview on the “normal” behavior of the infrastructure. If some systems start to act differently from this normal behavior, an attack is suspected and an alert is raised.

Whereas AECID (and similar tools) are detective measures (as they trigger alerts based on specific events that have happened already), our approach in the following is *preventive* in the sense of estimating and minimizing the risk of a successful APT from the beginning (cf. 1.2). Game theory is here applied to optimize the defense against a stealthy invader, who attempts to sneak into the system on a set of known paths, while the defender does its best to guard all these ways simultaneously. This is the abstract version of the situation that is normally summarized under the term APT.

Game theory appears as a natural tool to analyze conflicts of interest, such as obviously arise between the defender and the attacker mounting an APT. Powerful techniques to defend against stealthy takeover have been defined (partially originating from [16, 17] but also based on a variety of precursor and independent approaches, such as collected in [18]), but a method that fits into established risk management processes and can be instantiated with vague, fuzzy and qualitative risk assessments (such as uttered by domain experts) is demanding yet missing. Particularly intricate are matters of social risk response, say, if an enterprise seeks to minimize losses of reputation besides direct costs; assessing the public community’s response to certain actions being taken is a vague and difficult issue, to which sophisticated game theoretic [19–22] and agent based models [23] can be applied for an analysis and risk quantification. A recognized feature of any game-theoretic treatment of APT and in general every cyber-security scenario is the lack and asymmetry of information (say, the absence of knowledge about the attacker’s strategy spaces or payoffs, cf. [24, 25], while the attacker may have full information about the target system). This asymmetry is even stronger than what can be captured by many game-theoretic models, since organizational constraints may enforce the defender to act only at certain points in time, while the attacker is free to become active at any time. That is, the game is *discrete time* for one player, but *continuous time* for the other player—a setting that is hardly considered in game-theoretic literature related to security, and as such a central novelty in this work.

As we will show later (cf. Section 10.1 and Lemma 9), matrix games are nonetheless a proper model to account for what the *defender* can do against an APT, if we confine ourselves with the goal of playing the game to the best of our own protection and allow the outcomes to be random and unpredictable. Under this relaxation over the conventional game theoretic modelling, we can account for the outcome to be dependent on an action that is taken at different points in time, and especially also for actions that were interrupted before they could carry to completion. This addresses the issue identified by [26], who pointed out that moves may take a variable amount of time rather than being instantaneous (and thus atomic).

Ultimately, a significant obstacle for practitioners in the application of any game theoretic model is the lack of understanding of the ingredients to the game. That is, no matter how sophisticated the model may be, it nevertheless needs to be instantiated with whatever data is available. In many cases, this data is either qualitative (fuzzy) expert knowledge (formulated in some taxonomy, e.g., [27]) or obtained from simulation (see [28] for one example). Either may not be suitable to instantiate the proper APT model, even though the APT-game model would be quite sophisticated and powerful (such as [29]) in its capabilities for risk mitigation. In any case, this takes us to *empirical game-theoretic models*; a category into which this work falls.

### 1.2 Our Contribution

We present a novel form of capturing payoff uncertainty in game theoretic models. We deviate from standard games in the conceptual way of measuring the outcome of a gameplay not in crisp terms, but by an entire probability distribution object. That is, we play game theory on the abstract space of distributions for the following reasons:

1. The specification of losses and payoffs in a game is often difficult: how would we accurately quantify the results of a defense in light of an attack? Do we count the number of infected machines (such as done in [16])? Shall we work with monetary loss (causing difficulties in how to “price-tag” loss of reputation or consumer’s trust)? Conversely, can we play games over a categorical scale of payoffs, such as risk is being quantified in many standards like ISO 31000 [30] or similar?
  We can elegantly avoid any such issue by letting the game being defined by any outcome that can be ordered (as in conventional game theory), but in addition, allowing an action to have many different random outcomes (this is usually not possible in standard games). In doing so, we gain a considerable flexibility and degree of freedom to tackle a variety of issues, which we will discuss later on.
2. There is a strong asymmetry in the player’s information in many senses: first, the game structure itself is not common knowledge, since the defender knows only little about the opponent, while the opponent knows very much about the defender’s infrastructure (as lies in the nature of APTs, since these typically include an a-priori phase of investigation and espionage). Second, the game play is different for both players, since moves are not mutually observable, nor must happen instantaneously or even at the same times.
  Again, this can be captured by letting the effects of action be nondeterministic and random even if both, the attacker’s and defender’s action were both known.
3. Any game-theoretic model for security may itself be only part of an outer risk management process, and as such must be “compatible” with the surrounding workflows, which cover APT mitigation among other aspects. That is, the game theoretic model’s input and output must be useful with what the risk management process can deliver and requires. Our APT-games will be designed to fulfil this need.
4. Conventional stochastic models like Bayesian games indeed also capture uncertainty, but do so by letting the modeler describe a variety of different possible game structures, among which nature chooses at random in the actual gameplay. While different such structures can embody different outcomes, and the likelihood for these can be specified as a distribution (similar to what we do), each of these possible game structures must be specified in the classical way, thus effectively “multiplying” the problems of practitioners (if one game is difficult to specify, the specification of several ones does not appear to ease matters). Our approach avoids these issues by working with empirical data directly, and keeping the game models simple at the same time.

In light of the last point in particular, we will restrict our attention in the following to the problem of how to define games over qualitatively assessed outcomes that may be random. That is, the central question that this work discusses is essentially a form of reasoning under uncertainty:

**Given some possibilities to act, what would be the best choice if the consequences of an action are intrinsically random?**

We will show how to answer this question if the randomness can be modeled in the most general form by specifying probability distributions for the outcome. However, unlike normal optimization that maximizes some numeric quantity derived from the distribution of a random variable *X*, our games will optimize the *shape* of *X*’s distribution itself.

The presentation will heavily use examples for illustration, yet the concepts themselves will be described and also defined in a general form. To get started, consider the following example of decision making under the setting that we consider. Example 1 is about the protection of intellectual property rights (IPR), whose theft can be a reason to mount an APT.

**Example 1** (Assigning IPR Responsibilities). *Assume that an enterprise runs a project and is worried about protection of IPR. To mitigate this issue, one or more persons shall be put in charge of IPR protection. For this, say, three options are available:*

1. Assign IPR responsibility to one person: *This will increase the workload of the employee, and must be made w.r.t. available resources and skills. Neither is precisely quantifiable nor may be sufficient at all times. Thus, even assuming a strong commitment of the person to its role, some residual risk of damage occurring remains (human error of subordinates cannot be ultimately ruled out despite any strong supervision)*.
2. Assign IPR responsibility to a team of two or three persons: *Resources and skills may be much richer in this setting, but there is a danger of mutual reliance on one another, such that in the worst case, no-one really does the job (as a result of social coordination failure). Chances for this worst case to occur may be even higher than for option 1*.
3. Do security/ IPR training sessions: *here, we would completely rely the joint behavior of the employee’s and their commitment to the confidentiality of project content and adherence to the training’s messages. Nevertheless, chances for IPR loss due to human error (e.g., an unencrypted email leaking confidential information, or similar) may be lowered only temporarily, so that the training would have to be repeated from time to time*.

The optimal choice in Example 1 is not obvious, since consequences are not all entirely guaranteed for always foreseeable. Intuitively, we would go with the setting under which loss of intellectual property is least likely. Later, in Section 4, we will construct an ordering relation ⪯ that does exactly this if the loss distributions are defined on a scale of damage whose maximum is the loss of intellectual property (e.g., quantified by the business value attached to it). So, if we are somehow able to model possible random outcomes in each of the three scenarios, we can (algorithmically) compute the “best” (i.e., ⪯-minimal) loss distribution to be the best choice among the three above. This is a matter of loss distribution specification, which we will discuss in Section 5.1.

Finally, we remark that all of the theory sketched here has been implemented in R (including full fledged support of multi-criteria game theory based on distributions as discussed in section 8.1), to validate the method and to compute the results for the examples (such as in Section 9) shown here.

### 1.3 Organization of the paper

Section 2 briefly introduces the tools and concepts that our models are built upon. We will strongly rely on TVA (see Section 2.1) and human expertise in our model building, which we believe to be a viable approximation of how security risk management works in practice. Section 3 presents an example, which we will carry through the article to illustrate the concepts and approach as a whole. Section 4 introduces the theory of how decisions can be made if their outcome is rated through an entire probability distribution object (rather than a number), and Section 5 takes this basis to define games, equilibria and to highlight similarities but also an important qualitative difference between the so-generalized games and their classical counterparts. Sections 6 and 7 apply the framework to APTs by picking up the example from Section 3, and give algorithmic details on how to practically work out results (the aforementioned differences between our and classical games call for various mathematical tricks here). Section 8 briefly discusses generalizations towards multi-criteria decision making. Section 9 finishes the example from Section 3 by presenting results and security protection advices obtained from our game-theoretic APT mitigation game. Section 10 presents a critical discussion in terms of answering direct questions that were collected from practical experience with the proposed method in a research project (see the acknowledgment section at the end of the paper). Conclusions are drawn in Section 11.

## 2 Preliminaries and Notation

Vectors and matrices will be denoted as bold-face letters in lower case for vectors and upper case for matrices. *n* × *m*-Matrices over a set *M* are denoted as $A\in M^{n\times m}$, and the symbol ${\left(a_{n}\right)}_{n\in N}={\left(a_{n}\right)}_{n=1}^{\infty }$ is a shorthand for sequences. We use upper case normal font letters like *X* to denote random variables (RVs), and write *X* ∼ *F* to express that the RV *X* has the distribution function *F*. The respective density belonging to *F* is the corresponding lower case letter *f*, and where necessary, we add the subscript *F*_*X*_ or *f*_*X*_ to indicate the related RV for the density or distribution. For a given (finite) set *M*, we let S(M) be the set of all discrete probability distributions (the simplex) over *M*. Likewise, all families of sets, RVs or distribution functions are denoted in calligraphic letters (such as U,S or F). Estimates of a value are indicated by a hat, such as $\hat{F},\hat{f}$, to mean empirical distributions (normalized histograms). Approximations of an object *x* or *F* (scalar, distribution, etc.) are marked by a tilde, e.g., $\tilde{x},\tilde{F}$.

### 2.1 Topological Vulnerability Analysis

Topological vulnerability analysis [31] is the systematic identification of attacks to a system, based on the system’s structure and especially its network topology. The process usually consists of creating a complete picture of the infrastructure augmented by all available details about the components. Modeling the system’s topology as an (undirected) graph *G*(*V*, *E*) with a designated target node *v*_0_ ∈ *V*, we can use standard path searching algorithms to identify paths from the exterior of *G* towards the target node *v*_0_. Whatever structure is dug up by the TVA, an immediate question concerns the applicability of known attack patterns to the infrastructure model (similar to virus patterns being looked up in software). Graph matching techniques (see [32, 33] for example) appear as an interesting tool to apply here. The known (or suspected) vulnerabilities/exploits related to the nodes in *V* then determines which paths are theoretically open to *v*_0_ to successfully attack the system. These *attack paths* are thus sequences of vulnerabilities (augmented with the respective preconditions to exploit a vulnerability), and are the main output of a TVA. An APT can then (in a slightly simplified perspective) be viewed as the entirety of attack paths, and is physically mounted by sequentially working along a chosen attack path in a way that avoids detection of the attack at all stages (stealthy). Particular practical risk arises from exploits of not yet known vulnerabilities, which are commonly called *zero-day exploits*. Uncertainty about these partially roots in the complexity of the network, so that graph entropy measures (see, e.g., [34]) may be considered as a measure to help quantifying the chances of attacks coming over paths that were missed during the analysis. The practical handling of this residual risk is often a matter of using domain knowledge, collecting expert opinions, experience and information mining, combined with suitable mathematical models (e.g., [35, 36]). Our model immanently includes a zero-day vulnerability measure, as will be discussed in Section 7.2.

### 2.2 Attack Graphs and Attack Trees

**Fig 1:**
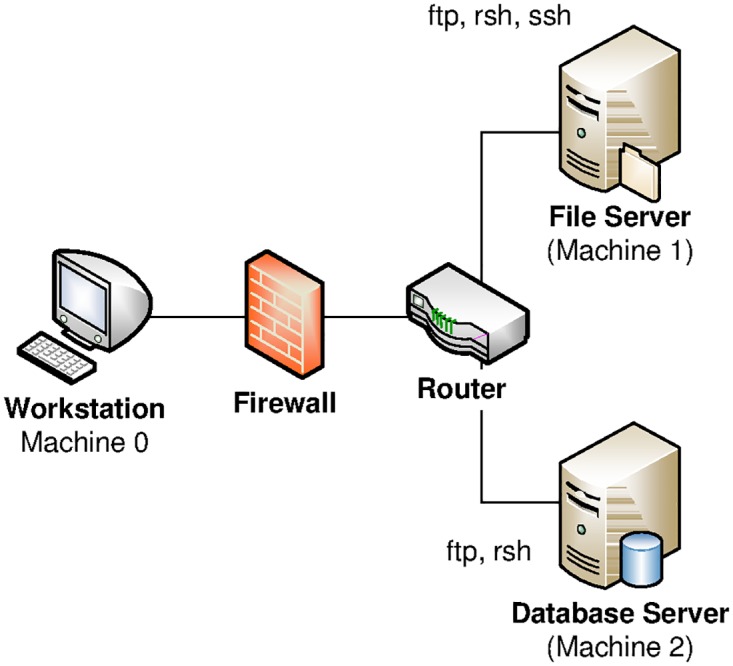
Infrastructure from [38] to illustrate game-theoretic APT modeling.

**Fig 2:**
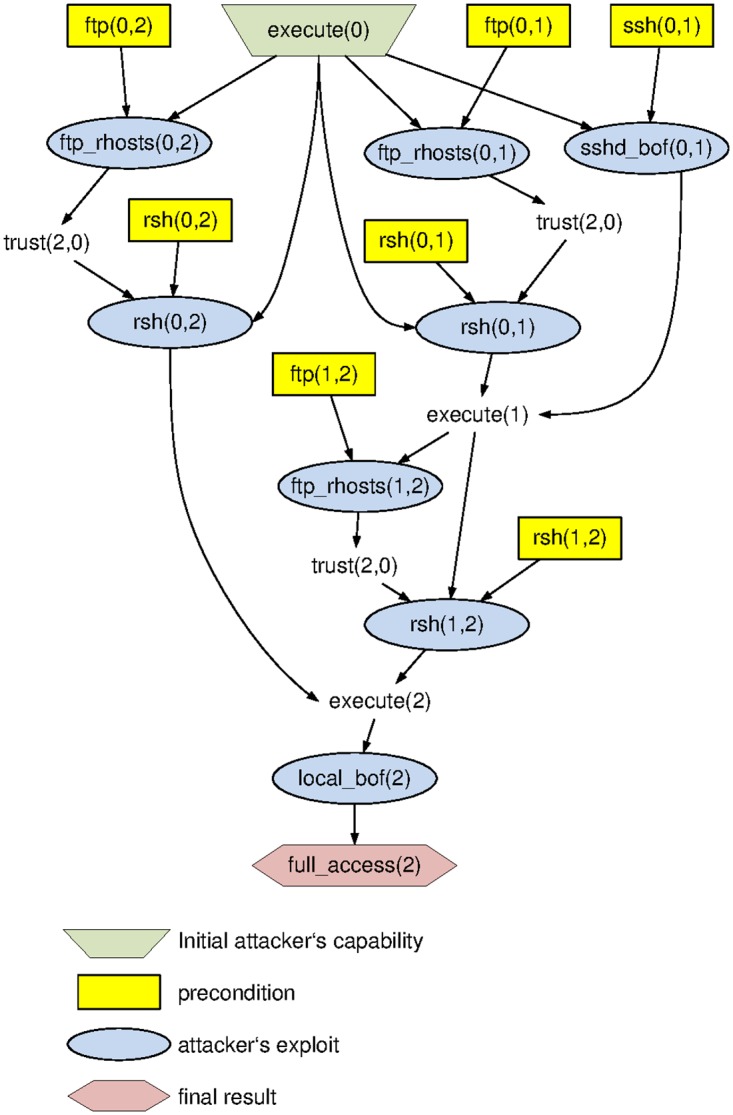
Example Attack Graph [38].

The entirety of ways into a system, including intersections and alternative routes on attack paths makes up the *attack graph* [37]. It is essentially a representation based on the system topology *G*, in which outgoing links of a node *v* are retained only if some exploit on *v* enables reaching *v*’s neighbor (see Figs 1 and 2 for an example). In terms of representation, an attack graph is to be distinguished from an attack tree, which is usually an AND/OR tree representing the possible exploit chains in a different way. Regardless of which is available, the main object of interest for our purposes is the set of attack paths, which directly corresponds to the action set of player 2 in our APT-games.

### 2.3 Extensive Form Games

Towards a game theoretic model of APT, we will use extensive form games (EFGs). While a full fledged formal definition of EFG is lengthy and complex, it will suffice to give a description of it to highlight the similarities to APTs. Formally, EFG are described by a tree *T*(*V*_*T*_, *E*_*T*_), with a designated root node that represents the starting stage in the game. Consequently, *T* has edges directed outwards from the root. For an APT, the root corresponds to the (hypothetical) point representing the exterior of the network graph *G*. The EFG is played between a set of (for our purposes two) players, including a hypothetical player “chance” that represents random moves in the game. Each node *v* ∈ *V*_*T*_ in the game tree *T*(*V*_*T*_, *E*_*T*_) carries an information on which player is currently at move (including the “chance” player). Furthermore, moves that are indistinguishable by other players are collected in a player’s *information set*. This, from the opponent’s perspective, represents the uncertainty about what a player has currently done in the game. In an APT model, the information set would correspond to possible locations where the attacker could currently be (again, recalling that an APT is stealthy). The EFG description is completed by assigning a vector of outcomes to the leaf nodes in the game tree. Normally, the outcomes are real values and specified for all players. Viewing an APT as an EFG, we would thus require to specify our own damage when the APT has been carried to the end (i.e., the target node *v*_0_ has been reached), but also the payoff to the adversary would needed to be known. The latter is a practical issue, since the uncertainty in the game is not only due to the attacker’s moves themselves, but also caused by external influences outside any of the player’s influences. Shifting all this uncertainty induced exteriorly to the chance-node in the EFG description appears infeasible, since much of it may depend on the particular action and current (even past) moves of both players (defender being player 1 and the attacker being player 2 in an APT game). However, given that the two players have different information on the current stage of the game play (only the attacker knows its precise position, the defender knows nothing; not even the presence of the attacker is assured), defining the information sets appears hardly doable.

Since the concept of EFG as being games with imperfect information essentially rests on information sets, any best behavior in the game play will inevitably rest on hypotheses on the player’s moves. For an EFG, we would describe these hypotheses as probability distributions on the information sets. In lack of these, we can only model the outcome based on the known defender’s actions and assuming the attacker to be possibly everywhere in the system. Practically, these hypotheses will rarely be available as hard figures and mostly come in qualitative terms like “low”, “medium” or “high” risk. Classical game theory is not naturally designed to work in such fuzzy terms.

Finally, an assumption of frequent criticism concerns the game model to be *common knowledge* to both players. This is certainly questionable in APT scenarios. In fact, a defender may in practice only have limited and widely uncertain information about the attacker’s incentives, current moves, current location or even its presence in the game. Thus, the information set would in the worst case cover the entirety of the game graph, and neither is the payoff to the attacker precisely quantifiable in most cases.

To avoid all these issues, we propose to replace the attacker’s payoffs by our own losses (in an implicit assumption of a zero-sum competition), in which an equilibrium behavior is a provable bound (see Lemma 9) to the payoff for the player having modeled the game. Second, we avoid difficulties of uncertain payoffs by defining the game play itself as a one-shot event, in which both players choose their strategies for the round of the game and the payoff is determined by that choice (thus, shifting all matters of uncertainty about where a player is in the game entirely to the payoffs). To this end, we will model an APT as a game with complete information but uncertain payoffs. In fact, the payoffs will be entire probability distributions rather than numbers.

## 3 A Running Example

Throughout this work, we will illustrate the steps and concepts using a running example borrowed from [38]. This reference describes a simple version of TVA (see, e.g., [31]) and attack graph modeling, based on a small infrastructure that is shown in Fig 1: The system consists of three machines (numbered as 0, 1 and 2), with several services being open on each node (such as file transfer protocol (FTP), remote shell (RSH) and secure shell (SSH)). The adversary attempts to gain access to machine 2, hereafter denoted as (the predicate) full_access(2). Towards its goal, the attacker may run different exploits from various points in the network, such as:

- FTP- or RSH-connections from a node x to a remote host y, hereafter denoted as ftp_rhosts(x, y), and rsh(x, y), respectively.
- a secure shell buffer overflow at node y, remotely initiated from node x, hereafter denoted as sshd_bof(x, y).
- local buffer overflows in node x, hereafter denoted as local_bof(x).

The actual APT is the attempt to use these exploits (and combinations thereof) in a stealthy fashion to penetrate the entire system towards establishing full access to the target machine 2. Naturally, exploits of any kind are subject to preconditions holding on the machine from which the exploit is initiated. We denote such a precondition on machine x to target a machine y in predicate notation as ftp(x, y), rsh(x, y) and ssh(x, y), w.r.t. the protocol being used. Depending on which services are enabled and responsive on each machine, a TVA can then be used to compile an attack graph (see Eq 2), which roots at the initial condition of having execution privileges on machine 0, denoted as execute(0), from which attacks can be mounted under the relevant preconditions. A particular APT scenario can be viewed as a path in the graph that starts from the root (execute(0)) via trust relations established between connected machines x and y (denoted as trust(x, y)), until the goal (full_access(2)).

Running the plain task of computing and enumerating all paths in the attack graph from execute(0) to full_access(2) digs up 8 attack vectors in our example. Each of these corresponds to one particular APT scenario, and the entirety of which makes up the adversary’s *action set*, denoted as *AS*_2_ (the subscript is used for consistency with the subsequent game theoretic model, in which the attacker is player 2. The defender will be player 1, respectively).

The next step in the risk mitigation process is the derivation of countermeasures from the identified attacks, such as, for example, the deactivation of services (to violate the necessary preconditions), or a patching strategy (to remove buffer overflow vulnerabilities), to name only two possibilities. Alas, none of these precautions is guaranteed to be feasible or even to work, as for instance:

- services may be vital to the system, say, deactivating an FTP connection may render the service offered by machine 1 useless.
- patches may work against a known buffer overflow, but an unknown number of similar exploits may nonetheless remain (thus enabling zero day attacks upon vulnerabilities found and offered for sale on the black market).

On the positive side, even unknown malware may be classified as such based on heuristics, experience or innovative antivirus technologies, all of which adds to the chances for the identified mitigation strategies to succeed. The practical issue here is, however, to deal with the residual risks and the inevitable uncertainty in the effectiveness of a protection. Ways to capture and handle these issues are theoretically described in Section 4 and applied to this example in Section 6.

For the time being, let us assume that a (non-exhaustive) selection of countermeasures has been identified and listed in Table 1. We call this list the *defender’s action set*, denoted as *AS*_1_ to indicate the defender as being player 1 in the subsequent APT game (Section 6). We leave this set incomplete here for the only sake of simplicity (in reality, the analysis would dig up a much richer set of countermeasures, such as can be based on the security controls catalog of relevant norms as ISO 27001 [39] or related).

**Table 1:** Security controls (selection).

Countermeasure	Comment
deactivation of services (FTP, RSH, SSH)	these may not be permanently disabled, but could be temporarily turned off or be requested on demand (provided that either is feasible in the organizational structure and its workflows)
software patches	this may catch known vulnerabilities (but not necessarily all of them), but can be done only if a patch is currently available
reinstalling entire machines	this wipes out unknown malware but comes at the cost of a temporary outage of a machine (thus, causing potential trouble with the overall system services)
organizational precautions	for example, repeated security trainings for the employees. These may also have only a temporary effect, since the security awareness is raised during the training, but the effect decays over time, which makes a repetition of the training necessary to have a permanent effect.

In general, the effect of an action, precaution, countermeasure, etc. is in most cases not deterministic and influenced by external factors beyond the defender’s influence and not even fully determined by the attacker’s actions. Furthermore, actions on both sides are usually not for free, and costs/losses on the defender’s side are induced by system outages (say, during a reinstall), staff unavailability (say, when people are in a training that itself may be costly), etc. Some of these costs may be precisely calculated, but others (say, if the system is offline during a reinstall) may depend on the current workload and thus be difficult to quantify.

Therefore, a *qualitative* risk assessment is often the only practical option (and an explicit recommendation by various standards such as ISO 31000 and by the German Federal Office of Information Security (BSI)). For a game theoretic analysis, however, this is inconvenient as it may result in a quite vague assessment of a countermeasure that may look like shown in Table 2.

**Table 2:** Example assessment of a security precaution.

Countermeasure: *patching*	
Aspect	Expert’s assessment
applicability	not always available
effectiveness	low or high (depending on the exploit)
cost	low to medium (e.g., if the system needs to be rebooted)

Similar assessments can be made for other protective measures as well, with quantitative figures occasionally being available (such as the costs for a security training, or the cost to install a new firewall or intrusion detection system). However, ambiguous and even inconsistent opinions may be obtained on the effectiveness and applicability of a certain action. Even if only one expert does the assessment in categorical terms as shown in Table 2, uncertainty may at least arise from none of the offered categories being appropriate for the real setting. That is, with “medium effectiveness” being a vaguely understood term in that context, an expert may utter a range of possibilities rather than confining her/himself to a specific statement. The example in Table 2 illustrates this by saying that the effectiveness of a patch can be either high (if the patch closes precisely the buffer overflow that was intended by the adversary), or even low, if the exploit has already been used to install a backdoor, so that the buffer overflow—even if it gets fixed—is no longer needed for the APT to continue. What is even worse, both assessments are at opposite ends of the scale (low/high), and can both be justified, thus telling hardly anything informative in this case.

**Fig 3:**
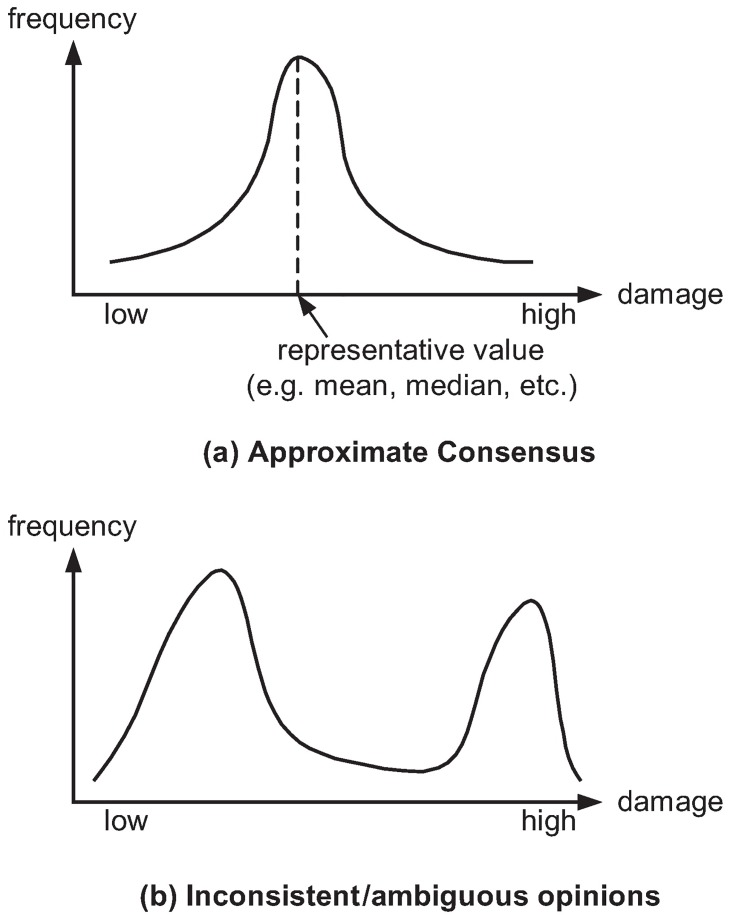
Agreeing vs. disagreeing expert ratings.

It is this point, where further opinions should be sought, which naturally will create a number of different assessments, some of which may be even mutually inconsistent (see Fig 3 for an illustration of how different opinions may accumulate at different points on the risk scale).

All this hinders the application of conventional decision or game theory, since in either approach (game or decision theoretic), we require a reasonably measurable effect for an action, and also a way to uniquely rank (order) different effects when a “best” action is sought.

## 4 Modeling Uncertainty for Decision-Support

If the outcomes of an action are uncertain, even random, then the most powerful model to express these would be to:

- collect as much data, expert opinions, etc. as is available,
- and compile a probability distribution from the available data, to capture the uncertainty in the assessments. Though this preserves all available information in the distribution object, the issue of *working* with it is more involved and a central technical contribution in this work.

In the best case, the assessments turn out to be quite consistent, with only a few outliers, in which case we may be able to define a reasonable representative (say, the average assessment; see Fig 3a). In other cases, however, the distribution may be multimodal, with each peak corresponding to different answers that may all have their own justification as being plausible (Fig 3b shows an example of many experts agreeing on either low or high effectiveness of the patching strategy).

**Fig 4:**
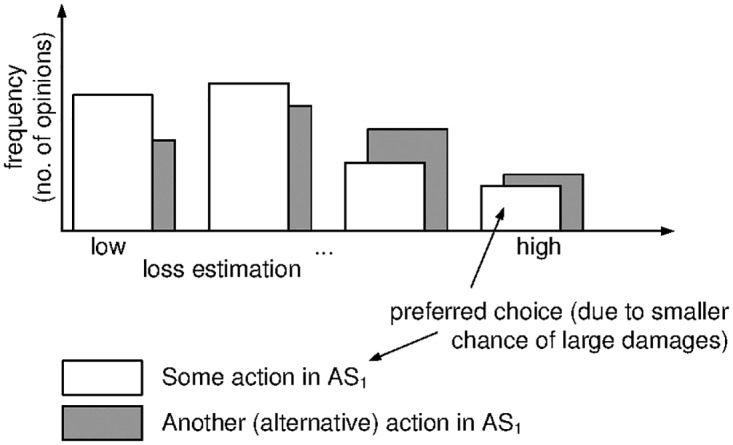
Comparing Different Preference Rules.

Finding a best action is typically done by assigning a utility value $u:\;AS_{1}\;\times \;AS_{2}\to \;\mathbb{R}$ (see Section 2.2 in [40]) to the actions to choose from *AS*_1_, *AS*_2_, and looking for the maximal such utility for both sides (defender and attacker). In quantitative risk management, a popular choice for this utility value is the expected damage, computed as

risk=damage×likelihood, (1)

which enjoys wide use throughout the literature (e.g., the ISO 31000 [30] or ISO 27000 [41] family of standards). This convention is easily recognized as being the first moment of some (usually not explicitly modeled) payoff distribution, and as such, is not satisfying in practice, as the mean tells us nothing about possible variations about it (Fig 4 illustrates the issue graphically). So, the variance would be the next natural value to ask for in addition to Eq (1). Continuing this approach, we can describe a distribution more and more accurately by using more and more moments, and indeed, the mapping

$$\phi :F\mapsto \ell ={\left(E\left(L^{n}\right)\right)}_{n\in N}\in R^{\infty }$$

provides a bijective link between a distribution function *F* and a representative infinite sequence of real numbers, provided that all moments exist. In a simple use of this representation, we could just lexicographically compare the sequences, letting the first judgement be based on the mean, and in case of equality, compare the variances, and so on. Such an ordering, however, appears undesirable in light of easy to construct examples that yield quite implausible preferences. Fig 4 shows an example.

An approach that preserves all information is treating the moment sequence as a hyperreal number $\phi (F)\in {}^{*}R$, so that we get a “natural ordering” on the distributions as it exists in the hyperreal space $\left({}^{*}R,\leq \right)$; see [42] for a full detailed treatment, which we leave here as being out of the scope of this work.

Nevertheless, it is important to recognize that the trick of embedding a distribution in the ordered field $\left({}^{*}R,\leq \right)$ of hyperreals equips us with a *full-fledged arithmetic* applicable to random payoff distributions, as well as a *stochastic ordering*, so that “optimality” of decisions can be defined soundly (later done in Definition 3). This implies that many well known and useful results from game and decision theory remain applicable in our setting (almost) as they are. Essentially, this saves us the labour of re-establishing a lot of theory, as would be necessary if another stochastic order (such as one in [43]) would be used.

Definition 2 will suitably restrict the class of loss distributions to ensure that all moments exist. Before that, however, let us briefly recap where the loss distributions will come from:

Given the attack graph that describes all APT scenarios and treating it as an EFG game description, we apply the same conversion of an EFG into the normal form of the game, which is a matrix. Let *n* = |*AS*_1_|, *m* = |*AS*_2_| be the number of threat mitigation strategies and possible exploits, which correspond to the action sets of both players (paths through the infrastructure determined by the possible exploits; cf. Table 3). Whereas a classical game would be described as a real valued payoff matrix $A\in R^{n\times m}$, the outcome in the APT game is not deterministic and as such will be described by a matrix of RVs $A={\left(L_{ij}\right)}_{i,j=1}^{n,m}$. Each variable *L*_*ij*_ describes the random loss (effect) of taking mitigation strategy *i* relative to the unknown *j*-th move of the adversary.

**Table 3:** APT scenarios (adversary’s action set *AS*_2_, based on Fig 2).

1	execute(0) → ftp_rhosts(0,1) → rsh(0,1) → ftp_rhosts(1,2) → sshd_bof(0,1) → rsh(1,2) → local_bof(2) → full_access(2)
2	execute(0) → ftp_rhosts(0,1) → rsh(0,1) → rsh(1,2) → local_bof(2) → full_access(2)
3	execute(0) → ftp_rhosts(0,2) → rsh(0,2) → local_bof(2) → full_access(2)
4	execute(0) → rsh(0,1) → ftp_rhosts(1,2) → sshd_bof(0,1) → rsh(1,2) → local_bof(2) → full_access(2)
5	execute(0) → rsh(0,1) → rsh(1,2) → local_bof(2) → full_access(2)
6	execute(0) → rsh(0,2) → local_bof(2) → full_access(2)
7	execute(0) → sshd_bof(0,1) → ftp_rhosts(1,2) → rsh(0,1) → rsh(1,2) → local_bof(2) → full_access(2)
8	execute(0) → sshd_bof(0,1) → rsh(1,2) → local_bof(2) → full_access(2)

**Definition 2** ((Random) Loss). *A real-valued RV L is called a* (random) loss, *if the following conditions are satisfied*:

- *L* ≥ 1 *(this can be assumed w.l.o.g.)*
- *The support of L, being* $supp\left(L\right):=\bar{\left\{x\in \mathbb{R}:f_{L}\left(x\right)>0\right\}}$, *is bounded (where the bar means the topological closure)*.
- *L has a density f_L_ w.r.t. either the counting- or the Lebesgue-measure. In the latter case, we assume the density f_L_ to be continuous on its support*.

*Define the set of loss distributions* F *to contain all distribution functions related to random losses*.

### 4.1 Optimal Decisions if Consequences are Uncertain

Definition 2 assures that the density function *f*_*L*_ of any random loss *L* admits moments *E*(*L*^*n*^) of all orders n∈N, so that we get a well-defined condition for an ordering based on moment sequences:

**Definition 3** (⪯-Preference between Loss Distributions). *Let L*_1_ ∼ *F*_1_, *L*_2_ ∼ *F*_2_ *be losses with* $F_{1},F_{2}\in F$. *We prefer L*_1_ *over L*_2_, *written as L*_1_ ⪯ *L*_2_, *if there is an index* $k_{0}\in N$ *so that* $E\left(L_{1}^{k}\right)\leq E\left(L_{2}^{k}\right)$ for all *k* ≥ *k*_0_. *We synonymously write F*_1_ ⪯ *F*_2_ *whenever we explicitly refer to distributions rather than RVs*.

A minor technical difficulty arises from the yet unsettled issue of whether or not there are non-isomorphic instances of $\left({}^{*}R,\leq \right)$, in which case we could get ambiguities in the ⪯-ordering. The next result, however, rules out this danger.

**Proposition 4** (cf. [44]). *The set* (F,⪯) *with* F *as in definition 2 and ⪯ as in definition 3 is a totally ordered set, where F*_1_ ⪯ *F*_2_ *implies* *ϕ*(*F*_1_) ≤ *ϕ*(*F*_2_), *with the embedding* $\phi :F\to ({}^{*}R,\leq )$, *and the ⪯-ordering on* F *is invariant w.r.t. how* $\left({}^{*}R,\leq \right)$ *is constructed*.

While the theoretical definition is easy, important practical questions about this preference demand an answer, in particular:

1. What is the practical meaning of the ⪯-ordering for risk management?
2. If ⪯ is practically meaningful, how can we (efficiently) decide it?

Let us postpone the answer to the first question until Section 4.3, and come to the algorithmic matters of deciding ⪯ first. The answer to the second question will then also deliver the answer to the first one.

### 4.2 Practical Decision of ⪯-Preferences

Let us first discuss the case where the loss distribution is continuous. Common examples in risk management (cf. [45]) are extreme value distribution or stable distribution (with fat tails). Although such distributions may not necessarily have a bounded support (thus not corresponding to a random loss in the sense of definition 2), we can approximate the distributions by random losses via defining a *risk acceptance threshold* 1<a∈R, and truncating the distribution outside the range [1, *a*]. The concrete value *a* can be chosen upon a desired accuracy *ε* > 0, for which we can choose *a* large enough to have the residual likelihood of damage > *a* is smaller than *ε*, or formally, Pr(*L* > *a*) < *ε* (we will come back to the choice of *a* in section 10.2).

Practically, the risk acceptance threshold is the value above which risks are simply “being taken” or are covered by proper insurance contracts. Thus, specifying the value *a* and truncating the loss distributions accordingly makes distributions with fat and/or unbounded tails fit as approximate versions into definition 2.

If *L*_1_, *L*_2_ have the same compact support [1,a]⊂R, and since the respective density functions *f*_*L*_1__, *f*_*L*_2__ are assumed continuous, both admit limits *b*_1_ = lim_*x* → *a*_ *f*_*L*_1__(*x*) and *b*_2_ = lim_*x* → *a*_ *f*_*L*_2__(*x*). For the moment, assume *b*_1_ ≠ *b*_2_, i.e., *f*_*L*_1__(*a*) ≠ *f*_*L*_2__(*a*) (the case of equality is treated later). The continuity of both functions implies that *f*_*L*_1__(*x*) ≠ *f*_*L*_2__(*x*) holds in an entire left neighborhood (*a* − *ε*, *a*] of *a* for some *ε* > 0. It is then a simple matter of calculus to verify that (since both *L*_1_, *L*_2_ ≥ 1), the speed of divergence of the respective moment sequences ${\left(E\left(L_{1}^{n}\right)\right)}_{n\in N}$ and ${\left(E\left(L_{2}^{n}\right)\right)}_{n\in N}$ is determined by which density function takes larger values in the region (*a* − *ε*, *a*], recalling that both densities vanish at *x* > *a*. That is, we have

$$\lim_{n\to \infty }\left[E\left(L_{1}^{n}\right)-E\left(L_{2}^{n}\right)\right]\in \left\{-\infty ,+\infty \right\},$$ (2)

and the condition of Definition 3 is ultimately satisfied (in either way).

**Lemma 5**. *Let L*_1_, *L*_2_ *be two random loss variables with continuous distribution functions* $F_{1},F_{2}\in F$, *and let f*_1_, *f*_2_ *denote the respective densities. If both RVs are supported in an interval [1, a] for* a∈R, *and there is some ε > 0 such that f*_1_(*x*) < *f*_2_(*x*) *for all x ∈ (a − ε, a], then L*_1_ ⪯ *L*_2_.

Lemma 5 (see [44] for a proof) offers an easy way to decide preferences based on the RV’s density functions only. The procedure is the following: Call [1, *a*] the common support of both loss variables *L*_1_, *L*_2_, and consider the density functions *f*_1_, *f*_2_:

- If *f*_1_(*a*) < *f*_2_(*a*), then *L*_1_ ⪯ *L*_2_,
- Otherwise, if *f*_1_(*a*) > *f*_2_(*a*), then *L*_2_ ⪯ *L*_1_.

Upon a tie, i.e., *f*_1_(*a*) = *f*_2_(*a*), we need to either decrease *a*, truncate the distributions properly, and repeat the analysis, or we may look at derivatives at *a* to tell us which density takes larger values locally near *a*. The latter approach is further expanded in Section 7.1.

**Fig 5:**
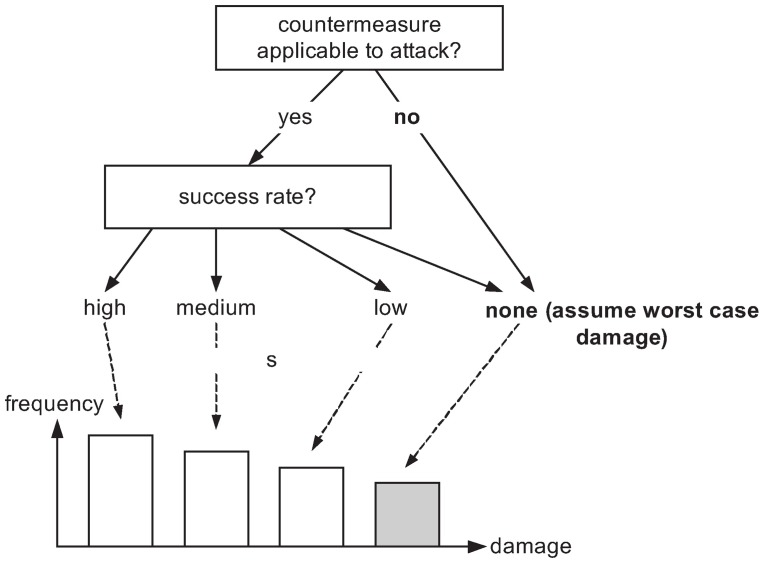
Example of ⪯-choosing among two empirical distributions (inconsistent expert opinions).

If the distribution is discrete, say, if the available data is not continuous but qualitative (e.g., categorical), then things are even simpler: if *L*_1_, *L*_2_ are both distributions over the same categories, then *L*_1_ ⪯ *L*_2_, if *L*_1_ puts less likelihood to categories of large damage than *L*_2_ (see Fig 5 for an example).

Formally, ⪯ thus boils down to a humble lexicographic ordering whenever the losses have categorical distributions.

**Definition 6** (lexicographic ordering). *For two vectors* $x=(x_{1},x_{2},\ldots )$ *and* $y=(y_{1},y_{2},\ldots )$ *of not necessarily the same length, we define* $x{<}_{lex}y$ *if and only if there is an index i_0_ so that x_i_0__ < y_i_0__ and x_i_ = y_i_ whenever i < i_0_*.

For two categorical distributions given in matrix notation and letting the support be given in descending order of risk levels *r*_*n*_ > *r*_*n*−1_ > … > *r*_1_, we observe that $F_{1}⪯F_{2}\;\Leftrightarrow \;\left(p_{n},\ldots ,p_{1}\right)=p{<}_{lex}q=\left(q_{n},\ldots ,q_{1}\right)$, when the distributions are:

$$F_{1}:\left(\begin{array}{ccccc} p_{n} & & \ldots & & p_{1}\\ r_{n} & > & \ldots & > & r_{1} \end{array}\right),\;\text{and}\;F_{2}:\left(\begin{array}{ccccc} q_{n} & & \ldots & & q_{1}\\ r_{n} & > & \ldots & > & r_{1} \end{array}\right)$$

That is, the action with the higher likelihood of extreme damage is less favorable, and upon a tie (equal chances of large damages), the likelihood for the next smaller risk level tips the scale, etc.

### 4.3 Practical Meaning of ⪯-Preferences

Summarizing the previous discussion in concise form directly takes us to the practical meaning of ⪯-preferences:

*We have L*_1_ ⪯ *L*_2_, *if large damages (near the maximum a) are more likely to occur under L*_2_ *than under L*_1_.

This is just an intuitive re-statement of Lemma 5. However, and remarkably, the converse to it is also true, which is the following:

**Theorem 7** (Thm 2.14 in [44]). *Let L*_1_, *L*_2_ *be two RVs with distribution functions* $F_{1},F_{2}\in F$. *If L*_1_ ⪯ *L*_2_, *then a threshold x*_0_ *exists such that* Pr(L_1_ > *x*) < Pr(*L*_2_ > *x*) *for every x* ≥ *x*_0_.

Restating this intuitively again, Theorem 7 tells that:

*a ⪯-minimal decision among two choices with respective consequences L*_1_ *and L*_2_ *minimizes the chances for large damages to occur.*

This is exactly what we are looking for: Risk management is in many cases focusing on extreme events rather than small distortions (which the system’s “natural” resilience is expected to handle anyway), and the focus of the ⪯-relation to prefer distributions with lighter tails perfectly accounts for this. Having ⪯ as a total ordering with a practical interpretation as being “risk-averse”, this already addresses the simple case of decision making among finitely many choices (as discussed in the next Section 5).

## 5 Practical Decision-Making

Remembering our example of APT mitigation, suppose that as an initial attempt, we would consider the installation of permanent security precautions, such as (additional) firewalls, access controls, physical protection, etc. Moreover, organizational changes such as were discussed in example 1 may be under discussion. However, all of these may have uncertain effectiveness, but the ⪯-relation now helps out.

In general and abstractly, the decision problem and procedure is the following:

- A set of choices (e.g., security precautions) *d*_1_, *d*_2_, …, *d*_*n*_ ∈ *AS*_1_ is available (e.g., defense actions for APT mitigation), each of which comes with a random consequence/effect captured by random losses *L*_1_, *L*_2_,…, *L*_*n*_.
- By looking for the ⪯-minimum among the distributions of *L*_1_,…, *L*_*n*_, we can take an optimal decision under uncertainty.

An open issue so far is where to get the losses from, an issue that will be revisited several times throughout this paper.

### 5.1 Constructing Loss Distributions

The simplest approach to construct loss distributions that satisfy definition 2 is to either:

- collect as much data as is available, and compile an empirical distribution from it,
- or define the loss distribution directly based on expertise (say, if the action’s incurred loss has a known distribution), if this is possible.

The latter case may occur seldom in practice, unless the particular threat has been studied specifically (such as disaster management or value at risk calls for extreme value distributions, etc.), and the “adversary” is nature itself. Against a rational adversary such as business competitors, hackers, etc., threat intelligence and expertise is the fundament upon which loss may be measured. Often, this assessment is made in qualitative terms for several (good) reasons, such as:

- Human reasoning is neither numeric nor crisp, i.e., experts may find it simpler to give assessments like “high risk” instead of having to specify a hard figure.
- Numerical precision can create the illusion of accuracy where there is none. There are only few types of incidents on which reliable statistical data is available, and having huge amounts of data on APTs attacks on general cybersecurity incidents may be unrealistic (and also undesirable if the incidents concern oneself).

In practice, rating actions w.r.t. their outcomes is naturally a matter of expert surveys, with answers possibly looking like shown in Table 2. Collecting many such opinions and putting them together in an empirical distribution $\hat{L}$ about a precaution’s performance may give distributions whose shape is unimodal (if a consensus among opinions is found), or multimodal, if disagreeing opinions are reported. Whatever happens or whether or not the outcome looks like illustrated in Fig 3, the ⪯-preference relation now allows for an elegant deal with this kind of uncertainty.

### 5.2 Games and Equilibria

With the uncertain outcome in a scenario of defense *i* vs. attack *j* being captured by a (perhaps empirical) probability distribution *L*_*ij*_, and the complete set of distributions being totally ordered w.r.t. ⪯, it is a simple and straightforward manner to define matrix games and equilibria in the well-known way. For convenience of the reader, we give the necessary concepts and definitions here.

Let *AS*_1_, *AS*_2_ be the action spaces for player 1 and 2, respectively, with cardinalities *n* and *m*. Let $A={\left(L_{ij}\right)}_{i,j=1}^{n,m}$ be a matrix of RVs that are all supported on the same compact set Ω=[1,a]⊂R. Let *F*_*ij*_ be the distribution function of the random loss *L*_*ij*_. In each round of the game, the random outcome *R* is conditional on the chosen actions of player 1 and player 2, and has the distribution *R* ∼ *L*_*ij*_ if player 1 chooses action *i* ∈ *AS*_1_ and player 2 chooses action *j* ∈ *AS*_2_.

We consider randomized choice rules $p=\left(p_{1},\ldots ,p_{n}\right)\in S\left(AS_{1}\right)$ and $q=\left(q_{1},\ldots ,q_{n}\right)\in S\left(AS_{2}\right)$, i.e., the vectors **p**, **q** describe the likelihoods of actions being taken by either player. In that case, the random outcome has a distribution *R* ∼ *F* (**p**, **q**) computed from the law of total probability, which is

$$F\left(p,q\right)\left(r\right)=\mathrm{Pr}\left(R\leq r\right)=\sum_{i,j}\mathrm{Pr}\left(R\leq r|i,j\right)\cdot \mathrm{Pr}\left(i,j\right)=\sum_{i,j}F_{ij}\left(r\right)\cdot p_{i}\cdot q_{j},$$ (3)

assuming a stochastically independent choice of actions by both players. This is the *utility function* in case of random outcomes (note that Eq (3) is exactly the same formula as is familiar from matrix game theory). So, the actual gameplay is *not* about maximizing the average revenue (as usual in game theory), but towards optimally “shaping” the outcome distribution *F* (**p**, **q**) towards ⪯-minimality. That is, in a zero-sum competition, player 1 and player 2 seek to choose their actions in order to minimize/maximize the likelihood of extreme events. Speaking differently again, player 1 attempts to shift the mass allocated by the respective density *f* (**p**, **q**) towards lowest damages, whereas player 2 tries his best to shape the density *f* towards putting more likelihood on larger damages. This is the essential technical process of our game-theoretic APT risk mitigation strategies, whose optimality is that of (standard) game-theoretic equilibria in zero-sum matrix games (see [46] for a formal treatment).

As for standard games, it can be shown that the saddle-point value $V\left(A\right)=\max_{p\in S(AS_{1})}\min_{q\in S(AS_{2})}$ *F* (**p**, **q**)is invariant w.r.t. different equilibria, and that equilibria defined w.r.t. ⪯ exist (and can be generalized to Nash-equilibria in *n*-person games in the canonic way). The way of proving it makes use of the embedding of distributions into the hyperreal space ${}^{*}R$, where all the known results necessary to re-establish the fundament of game theory are available (yet further substantiating our loss representation by a moment sequence). Unfortunately, however, not all properties are directly inherited, such as a central computational feature of zero-sum games is absent in our setting:

**Proposition 8**. *There exist zero-sum matrix games* $A\in F^{n\times m}$ *for which fictitious play (according to* [47, 48]) *does not converge*.

Proposition 8 is proved by constructing a concrete example (see [49]) of a game that cannot be solved using fictitious play. Thus, it is an unfortunate obstacle in applying well-known game theory to our new setting. The formal fix relies on yet another representation of the loss densities, which admits converting a matrix game over F into a set of standard matrix games over R, which can be solved by fictitious play again. The details of this are postponed until Section 7, culminating in the main Theorem 14 that assures that we can ultimately escape the situation that proposition 8 warns us about. Interestingly, this fix has a useful side-effect, whose physical meaning is a heuristic account for zero-day exploits. We will revisit this aspect in more detail later in Section 7.2.

## 6 APTs as Games

Suppose that a TVA has been done and that an attack graph is available. Towards a game-theoretic model of APTs, let us think of the attack graph as sort of an extensive form game (EFG) with perfect information. Although the attack graph or tree may not follow the proper syntax of an EFG, we can nevertheless convert it into syntactically correct normal form game in the same way as we would do with an EFG. That is, we would traverse the graph from the initial stage of the game until the stage where payoffs are issued to all players. From the exhaustive list of all these paths, we can define the strategies of both players as rules about what to do at which stage, given the other player’s move. Likewise, an APT would in this view be mounted along any of the existing paths from the root of the attack graph down to the goal, with the difference to EFG mostly being the fact that the “game” does not clearly define when the players are taking their moves (this is a conceptual difference to EFG, where the assignment of which player’s move it is part of the EFG description).

In both cases, EFG and APT attack graphs, we can compile a set of paths from the start to the finish, from which strategies for both players can be identified. While this identification comes from the definition of the EFG, for APTs, the strategies are delivered only for the opponent player 2, which is the attacker. Player 1, the defender, needs to derive its action set *AS*_1_ based on player 2’s actions *AS*_2_. Table 4 summarizes the correspondence between EFG and APT attack trees.

**Table 4:** Correspondence of Attack Trees/Graphs and Extensive Form Games.

Extensive form game	Attack tree/graph
start of the game	root of the tree/graph
stage of the gameplay	node in the tree/graph
allowed moves at each stage (for the adversary)	possible exploits at each node
end of the game	leaf node (attack target)
strategies	paths from the root to the leaf (= attack vectors)
information sets	uncertainty in the attacker’s current position and move

The nature of APTs induces a difficulty in the game specification here, since we usually do not know how deep the attacker may have penetrated into the system, and because of this, the current stage of the game is expectedly unknown to the defender. Countermeasures against exploits in each stage may be identified, but not always possible, feasible or successful. Allowing for a random outcome with the possible event of an action to fail elegantly tackles this issue in our setting.

If countermeasures have no permanent effect or are likely to fail, then we may need to repeat them. For example, a security training may cause only temporarily raised security awareness. Likewise, updating a software once is clearly useless unless the system is continuously kept up to date.

Given that the defense actions in *AS*_1_ must be repeated, we can set up a matrix game to tell us the best way to do so. Since precautions cannot be applied everywhere at all times, we need to define the game as one where the defender takes random moves, based on a hypothesis where the attacker may currently be. Alas, it would probably not be feasible to rely on Bayesian updating towards refining our hypotheses, since this assumes much data, i.e., many incidents to occur, and hence is exactly what APT mitigation seeks to prevent. Thus, many popular tools from game theory like perfect Bayesian equilibria or related appear unattractive in our setting.

To simplify the issue in general, let us assume that the defender can access all parts of the system (thus, take moves related to any stage of the game as defined by the attack tree), whereas the attacker can move only from its current location (node) to the successor (child node) location. The game play is thus a matter of the defender seeking the optimal way of applying its threat mitigation moves anywhere at random in the infrastructure, in an attempt to keep the adversary away from its target. Unlike a Bayesian or sequential game approach, the application of defense actions is not based on hypotheses of where the attacker currently is, but will assume a worst-case behavior of the attacker (thus, switching from the Bayesian towards the minimax decision theoretic paradigm).

For example, applying a patch at some point may close a previously established backdoor and send the adversary back to the start again. However, if the patch is currently unavailable, not effective or simply applied to the wrong machine, the defense move will have no effect at all. Towards modeling this uncertainty, let us first become more specific on what the payoffs in the example game will be.

Following the common qualitative risk assessments, we may define categories of risk depending on “how far away” the adversary is from its destination in the attack graph. Collecting the lengths of all paths listed in Table 3, we see that their lengths range between 4 and 8 nodes (including the start execute(0) and finish node full_access(2)). In any such case, we may simply map the distances to qualitative scores, such as Table 5 proposes here.

**Table 5:** Possible mapping of graph distance to risk categories.

Distance	Risk
7…8	low
3…6	medium
0…2	high

The concrete mapping of distances to risk levels can, however, already induces uncertainty. For example, assume that an attacker has already gained execution privileges on machine 1 (denoted as execute(1) in the attack graph), then it may either continue its way on path 4 in Table 3 (via a remote FTP connection from machine 1 to machine 2; node ftp_rhosts(1, 2)) or on path 5 in Table 3 (via an RSH connection from machine 1 to machine 2; node rsh(1, 2)). On path 4, the distance to full_access(2) is 4 nodes (ftp_rhosts(1, 2) → sshd_bof(0, 1) → rsh(1, 2) → local_bof(2)), while on path 5, the distance is only 2 nodes (rsh(1, 2) → local_bof(2)). In light of the a-priori specified mapping of distance to risk levels as in Table 5, the risk would be classified as *either* “medium” or “high”, depending on which path has been chosen. The usual stealthiness of APTs hence causes uncertainty in the risk assessment, which needs to be captured by a proper decision- or game-theoretic APT mitigation approach.

### 6.1 Identifying Mitigation Strategies

Having the attack graph and once attack vectors have been derived from it, all of which are collected in the adversary’s action space *AS*_2_, the next step is the identification of mitigation strategies. This process is a standard phase in many risk management practices, and often based on known countermeasures against the identified threats (accounting for unexpected events is a matter of zero-day exploit handling, which we will revisit shortly in Section 7.2). Since the process of defining countermeasures is a task that highly depends on the attack vectors *AS*_2_, we cannot define a general purpose procedure to identify *AS*_1_ here (it is individual and different for various infrastructures). For the sake of generality and conciseness of presentation in this work, let us therefore assume that all relevant defense actions are available and constitute the action set *AS*_1_ for the defender. It may well be the case that not all actions are effective against all threats, and neither may a designated countermeasure *i* be necessarily effective against threat *j*. In that case, we may pessimistically assume maximal damage to be likely in scenario (*i*, *j*). Matching all defenses in *AS*_1_ against all attack vectors in *AS*_2_ is a matter of defining the game’s loss distributions, which is the next step towards completing the game model in Section 6.2. To simplify the notation in the following, let us abstractly denote the action spaces as *AS*_1_ = {1, 2, …, *n*} and *AS*_2_ = {1, 2, …, *m*}, with the specific details of the *i*-th defense and the *j*-th attack for all *i*, *j* being available in the risk management documentation (in the background of our modeling).

### 6.2 Defining the APT Game

**Fig 6:**
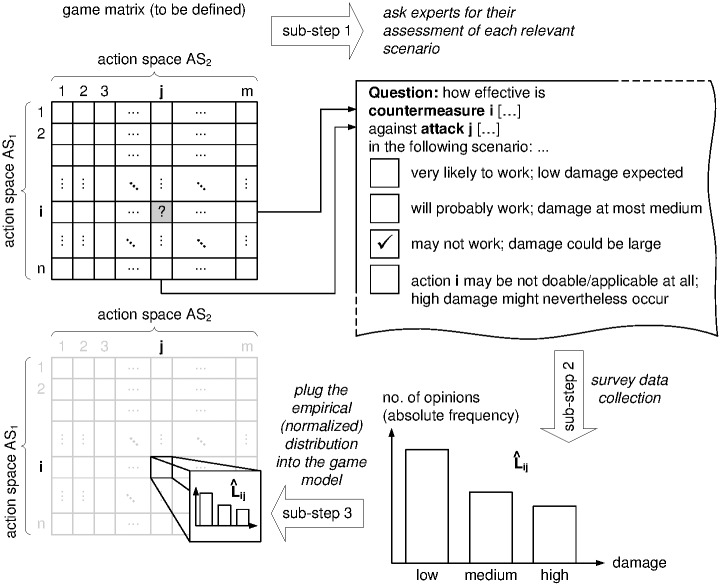
Loss Assessment of Counteraction vs. Threat.

Towards a matrix game model of APTs, it remains to specify the outcome of each attack/defense scenario. To capture the intrinsic uncertainty here, we will resort to a qualitative assessment like sketched above and/or arising from vague opinions like Table 2 illustrates. The APT game is then defined upon loss distributions according to definition 2 to describe the potential loss in each scenario (*i*, *j*) ∈ *AS*_1_ × *AS*_2_. In cases where we are unable to come up with a reasonable guess on the distributions, a pessimistic approach towards a worst-case assessment could work as follows (we will later revisit the issue in the discussion Section 10.2):

1. Fix a particular position in the network, i.e., a certain point where an attack is considered. Let the hypothesized attack be the one with index *j*_0_ ∈ *AS*_2_.
2. Fix a defense action *i* ∈ *AS*_1_.
3. In lack of better knowledge, assume a uniform distribution of all possible *j*, including *j*_0_, and rate the success probability *p*_*j*_0__ of the defense conditional on *j* = *j*_0_ (i.e., if your guess was right). This rating can also be made conditional on the expected “doability” of the current defense (e.g., if the defense means patching, the patch may not be available at all times, or it may be ineffective). Fig 6 displays the process as a decision tree, in which the worst case outcome (highlighted in gray) is taken if either the countermeasure is considered as possibly effective but may still fail (with a certain likelihood), or if the countermeasure is not applicable at all. In any case, the expert—based on the assumed attacker’s behavior—is not bound to confine her/himself to a single answer, and may rate all the possibilities with different likelihoods
4. If possible, collect many such assessments (say, from surveys, simulations, etc.), and compile an empirical distribution from the available data. This empirical distribution is then nothing else than a histogram recording the number of uttered opinions (shown as the bar chart in Fig 6) Note that the uniformity assumption on the attacker’s location can be replaced by a more informed guess, if one is available. For example, the adversarial risk analysis (ARA) framework [50–52] addresses exactly this issue.

**Fig 7:**
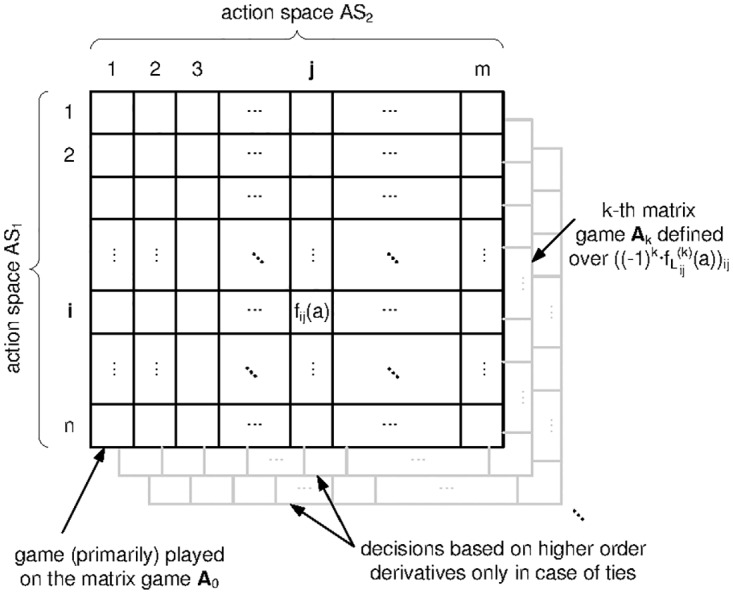
Specification of an APT Game (Example Workflow Snapshot).

This procedure is repeated for the entirety of scenarios in *AS*_1_ × *AS*_2_, i.e., until the full game matrix has been specified. Fig 7 illustrates the three sub-steps per entry of this procedure in the game matrix (note that the right-most decision path (bold-printed in Fig 6) is reflected as the fourth choice option in the example survey shown in Fig 7). We stress that the full lot of |*AS*_1_| ⋅ |*AS*_2_| may hardly be necessary to put to a survey, since not all actions are effective against one another (defenses may work against specific threats, so only a few combinations in |*AS*_1_| ⋅ |*AS*_2_| need to be polled explicitly, and others can rely on default settings; cf. Fig 6). Furthermore, some loss distributions can be equally well computed from simulations (cf., e.g., [53]).

By construction of the total ordering, the game that we define to minimize the loss would then be played towards minimizing the likelihood for large damages (by Theorem 7). Returning to our example sketched in Section 3, the uncertainty in a risk level quantification based on distance in the graph would thus mean that the gameplay is such as to keep the adversary “as far away as possible” from its target. This is indeed what we would naturally expect, and the ⪯-relation acting on loss distributions that are based on distance achieve precisely this kind of defense.

Although this modeling of APTs is heavily based on (subjective) expertise and manual labour, it fits quite well into standard risk management processes (such as ISO 31000 [30] or ISO 27005 [54]), and nevertheless greatly simplifies matters of modelling over the classical approach, as a variety of issues are elegantly solved. A selection is summarized in Table 6, with a complementary discussion given in Section 10.2. Additional help in the specification of risk assessments is also offered by thinking about costs of an exploit or known ratings of vulnerabilities such as by common vulnerability scoring system (CVSS) [55]. Such ratings are commonly delivered along with the TVA (e.g., by tools like OpenVAS).

**Table 6:** Benefits of Distribution-Valued Game-Modeling over Classical Game-Modeling.

Issue	Classical game-theoretic modelling	How this is handled in distribution-valued games
Payoff uncertainty	Either switching to special forms of equilibria (disturbed, trembling hands, etc.) or agreeing on a simultaneously representative value for all possible outcomes (“consolidation of different opinions”)	No consolidation or representation needed; we can simply work with the (normalized) histogram of all possible outcomes (or opinions on what could happen)
Non-realizable strategy	Separating out cases where a strategy can be played or not. This would amount to specifying two versions of the strategy (one that is successful and one that fails)	Since actions can by construction have many different outcomes, success and failure are just two realizations of the corresponding loss RV *L*, each of which may occur with a known (or estimated) probability. The entirety of these probabilities makes the sought density function *f* of the RV *L*.
Imperfect information	Working with hypotheses on expected moves in stages of the game where no precise information is available. The hypotheses can be learnt from past history and are taken into account when defining the optimal behavior (e.g., Bayesian perfect equilibrium)	Is directly incorporated in the uncertainty of the outcome, since an unknown move corresponds in a perceived random payoff; thus, there is no intrinsic conceptual difference here
Random changes in the game-play (stochastic games [56])	Resorting to special forms of equilibria, such as distorted or trembling hands equilibria or stochastic games [46, 56]	As long as the outcome remains identically (stationarily) distributed across several rounds of the gameplay, there is no specific treatment required upon random changes in the gameplay. The known theory of Markov chains can be used here to analyze the changes in the gameplay for stationarity.

## 7 Practical Computation of Optimal Defenses

Essentially, our APT game model is a matrix game $A\in F^{\left|AS_{1}|\times |AS_{2}\right|}$, in which each defense *i* ∈ *AS*_1_ vs. each attack in *j* ∈ *AS*_2_ is rated in terms of a probability distribution (uncertain outcome) $F_{ij}\in F$. By defining the losses in the gameplay to be the gain for the adversary (i.e., making the competition zero-sum), we obtain a valid worst-case approximation that enjoys the following useful property:

**Lemma 9**. *Let AS*_1_, *AS*_2_ *be the action spaces for the defender and the attacker, respectively, with cardinalities n and m. Furthermore, let* **B** *be the (unknown) matrix of true payoffs for the attacker, and let* **A** *be the loss matrix for the defender. If the saddle-point values of the zero-sum matrix game* **A** *is V* (**A**), *and* *V* (**A**, **B**) *is any equilibrium payoff in the bimatrix game induced by* **A**, **B**, *then we have*:

V(A,B)⪯V(A), (4)

*provided that the defender plays a zero-sum equilibrium strategy (induced by* **A**) *in both games, the zero-sum game* **A** *and the bi-matrix game* (**A**, **B**).

Lemma 9 directly follows from the definition of equilibria, and is a well known fact; cf. [18] for a more elaborate discussion.

Intuitively, Lemma 4 says that the worst case attack occurs when the adversary’s incentives are exactly opposite to our own interest. In particular, observe that the upper bound Eq (4) is independent of the adversary’s payoff/incentive structure **B**. The irrelevance of **B** in the upper bound tells that we do not require any information about the adversary’s intentions or incentives, as *V* (**A**) can be computed only based on the defender’s possible losses. Thus, in playing the zero-sum defense we obtain a baseline security that is guaranteed irrespectively of how the adversary behaves, provided it acts only within its action set *AS*_2_. The case of unexpected behavior, that is, actions outside *AS*_2_, corresponds to an unforeseen zero-day exploit. A remarkable feature of the game model using distribution-valued payoffs is its natural account for such outcomes, which we will further discuss in Section 7.2.

Our use of non-standard calculus somewhat limits the practically doable arithmetic, since for example, divisions in ${}^{*}R$ require an ultrafilter to fully describe ${}^{*}R$ (which is unavailable since ${}^{*}R$ is defined non-constructively; see [42]). Fortunately, however, these issues do not apply for our matrix games here, since Nash equilibria can still be computed by fictitious play (FP) [47, 48, 57] which uses practically doable ⪯-comparisons only. The unpleasant possibility of non-convergent FP (proposition 8 warns us about this) is escaped by using non-parametric (kernel density) models for the payoff distributions, and using Lemma 18 to decide ⪯. The respective details are laid out in section 7.1. This closes a gap left open in [58].

### 7.1 Computing Optimal Defenses (Equilibria)

To avoid proposition 8 to apply for our APT-games, we need to assure that all loss distributions share the same support (the counterexample used to prove proposition 8 relies on different losses whose supports that are strictly contained in one another). To this end, we will introduce another representation of a loss distribution as a sequence, so that the lexicographic ordering <_*lex*_ on the new sequence equals the ⪯-ordering of definition 3.

To make things precise, let us assume that a particular empirical loss distribution ${\hat{L}}_{ij}$ has been compiled from the data *x*_1_, *x*_2_, …, *x*_*N*_, as obtained from simulations or expert questionnaires. Moreover, assume that ${\hat{L}}_{ij}$ is categorical, so that the underlying data points (answers) can be ranked within a finite range (in the example in Fig 7, we have three categories, e.g., {“low”, “medium”, “high”}, which correspond to the ordered ranks {1, 2, 3}). The case when the loss distributions is continuous is in fact even simpler, and discussed later in remark 10. For now, let us stick with the expectedly more common practical case where risk assessments are made in categories rather than hard figures.

First, the empirical distribution (normalized histogram) ${\hat{L}}_{ij}$ is replaced by a kernel density estimator (KDE) using Gaussian kernels of a fixed bandwidth to define

$${\tilde{f}}_{{\hat{L}}_{ij}}\left(x\right)=\frac{1}{N\cdot h}\sum_{k=1}^{N}K\left(\frac{x_{k}-x}{h}\right),$$ (5)

where

- $K\left(x\right)=\frac{1}{\sqrt{2\pi }}\exp(-\frac{1}{2}x^{2})$ is the standard Gaussian function,
- and *h* > 0 is a bandwidth parameter that can be estimated using (any) standard statistical rule (of thumb, e.g., Silverman’s formula [59]).

Although any such nonparametric estimation can be quite inaccurate, yet as the data on which it is based is subjective anyway, the additional approximation error may not be as significant (nor in any sense quantifiable; still, Nadaraja’s theorem (see [60]) would assure that a continuous unknown underlying loss distribution would be approximated arbitrarily well in probability, as *N* → ∞, provided that *h* is chosen as *h*(*N*) = *c* ⋅ *N*^−*α*^ for any two constants *c* > 0 and 0 < *α* < 1/2.)

Using the KDE Eq (5), we can cast the distribution-valued game back into a regular matrix-game over the reals. Note that in choosing Gaussian kernels, we naturally extend all density functions to the entirety of R. Such distributions would not be losses in the sense of definition 2, as the supports are no longer bounded.

Using the aforementioned risk acceptance threshold *a* > 1 to truncate all loss distributions at *a*, casts the loss distributions into the proper form. We can then expand the (truncated) loss density $f_{{\hat{L}}_{ij}}$ into a Taylor series for every scenario (*i*, *j*) ∈ *AS*_1_ × *AS*_2_. To ease notation in the following, let us drop the double index and simply write $\tilde{f}$ to mean the kernel density approximation of the empirical distribution in the given scenario, based on *N* data samples. Then, its Taylor series expansion at point *a* is

$$\tilde{f}\left(x\right)=\sum_{k=0}^{\infty }\frac{{\left(x-a\right)}^{k}}{k!}{\tilde{f}}^{\left(k\right)}\left(a\right),$$ (6)

which converges everywhere on [1, *a*] by our choice of the Gaussian kernel. The *k*-th inner derivative is obtained from the kernel density definition Eq (5) as,

$$f^{\left(k\right)}\left(x\right)=\frac{1}{N\cdot h^{2}\cdot \sqrt{2\pi }}\sum_{j=1}^{N}\frac{d^{k}}{dx^{k}}\exp\left(-\frac{1}{2h^{2}}{\left(x_{j}-x\right)}^{2}\right)$$ (7)

Here, our use of Gaussian density pays a second time, since the *k*-th derivative of the exponential term can be expressed in closed form using Hermite polynomials by exploiting the relation

$${\left(-1\right)}^{k}\exp\left(\frac{x^{2}}{2}\right)\frac{d^{k}}{dx^{k}}\exp\left(-\frac{x^{2}}{2}\right)=2^{-\frac{k}{2}}H_{k}\left(\frac{x}{\sqrt{2}}\right),$$ (8)

in which *H*_*k*_(*x*) is the *k*-th Hermite polynomial, defined recursively as *H*_*k*+1_(*x*) ≔ 2*xH*_*k*_(*x*) − 2*H*_*k*−1_(*x*) upon *H*_0_(*x*) = 1 and *H*_1_(*x*) = 2*x*. Plugging Eqs (8) into (7), and after rearranging terms, we find

$$f^{\left(k\right)}\left(x\right)=\frac{1}{N\sqrt{\pi }}\frac{{\left(-1\right)}^{k}}{{\left(h\cdot \sqrt{2}\right)}^{k+1}}\times \sum_{j=1}^{n}\left[H_{k}\left(\frac{x-x_{j}}{h\sqrt{2}}\right)\cdot \exp\left(-\frac{{\left(x-x_{j}\right)}^{2}}{2h^{2}}\right)\right]$$ (9)

Evaluating the derivatives up to some order and substituting the values back into Eq (6), we could numerically construct the kernel density estimator. Fortunately, there is a shortcut here to avoid this, if we use the vector of derivatives with alternating signs to represent the Taylor-series expansion, and in turn the KDE, by

$${\tilde{f}}_{{\hat{L}}_{ij}}≃{\left({\left(-1\right)}^{k}f_{{\hat{L}}_{ij}}^{\left(k\right)}\left(a\right)\right)}_{k=0}^{\infty }=\left(y_{0},y_{1},y_{2},\ldots \right)\in R^{\infty },$$ (10)

where the entries of the sequence can be computed from Eq (9).

Interestingly, under the assumptions made (i.e., truncation at a point 1<a∈R and approximating the empirical distribution by a Gaussian KDE), the *lexicographic order* on the series representation Eq (9) *equals* the preference order ⪯ on the hyperreal representation of the loss distribution (see Lemma 18 in the appendix for a proof). That is, we can decide ⪯ between two sequence representations ${\left(y_{k}\right)}_{k=0}^{\infty }$ and ${\left(z_{k}\right)}_{k=0}^{\infty }$ of the form Eq (10) as follows:

- If *y*_0_ < *z*_0_, then *L*_1_ ⪯ *L*_2_. If *y*_0_ > *z*_0_, then *L*_2_ ⪯ *L*_1_. Otherwise, *y*_0_ = *z*_0_, and we check
- if *y*_1_ < *z*_1_, then *L*_1_ ⪯ *L*_2_. If *y*_1_ > *z*_1_, then *L*_2_ ⪯ *L*_1_. Otherwise, *y*_1_ = *z*_1_, and we check
- if *y*_2_ < *z*_2_, then *L*_1_ ⪯ *L*_2_, etc.

**Remark 10** (Continuous loss models). *If a continuous loss model is specified, differentiability may be not be an issue if the density f has derivatives of all orders. Otherwise, we can convolve f by a Gaussian density k_h_ with small variance h > 0 to get an approximation* $\tilde{f}=f*k_{h}\in C^{\infty }$ *at any desired precision. Kernel density estimates are exactly such convolutions and thus provide convenient differentiability properties here*.

**Fig 8:**
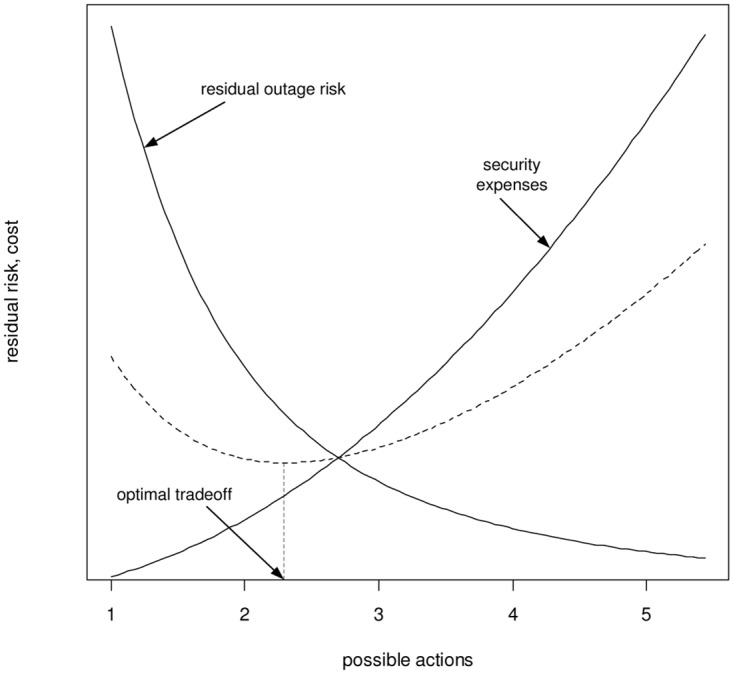
Applying Fictitious Play.

Experimentally, we observed that in many cases the preference decision ⪯ can be made already using the first value *f*(*a*) in the sequence Eq (10). If the decision cannot be made (upon a tie *f*_*L*_1__(*a*) = *f*_*L*_2__(*a*)), then we can move on to the first order derivative, and so on. Thus, we technically do fictitious play in parallel on a “stack” of matrix games $A_{0},A_{1},A_{2},\ldots \in R^{\left|AS_{1}|\times |AS_{2}\right|}$, where the *k*-th matrix is constructed (only on demand) with the *k*-th entry of the sequence representation Eq (10). The selection of strategies is herein always made on the first game matrix **A**_0_, looking at the others only in cases where the decision cannot be made directly within **A**_0_ (see Fig 8 for an illustration). Since we are now back at a regular matrix game, the usual convergence properties of fictitious play are restored.

Of course, we cannot run fictitious play on an infinite stack of matrix games, so we are necessarily forced to restrict attention to a finite “sub-stack”. However, depending on how “deep” the stack is made, we can reach an equilibrium at arbitrary precision. This is made rigorous in the following definition:

**Definition 11** (Approximate Equilibrium). *Let ε > 0, δ > 0 be given, and let* $A=F^{n\times m}$ *be a zero-sum matrix game with distribution-valued payoffs. We call a strategy profile* $\left({\tilde{p}}^{*},{\tilde{q}}^{*}\right)\in R^{n+m}$ *an (ε, δ)-approximate equilibrium, if there is an equilibrium* $\left(p^{*},q^{*}\right)\in R^{n+m}$ (*in the zero-sum game* **A**) *such that both of the following conditions hold*:

1. $\|\left(\tilde{p}{}^{*},\tilde{q}{}^{*}\right)\;-\;{\left(p{}^{*},q{}^{*}\right)\|}_{\infty }<\varepsilon$, and
2. $\|\tilde{F}{}^{*}\;-\;{F{}^{*}\|}_{L^{1}}<\delta$,

*where the equilibrium payoffs* ${\tilde{F}}^{*}$ *and* $F^{*}$ *are defined by* Eq (3) *upon the approximate and the (regular) equilibrium, respectively*.

**Remark 12**. *By the equivalence of norms on R, the ∞-norm in condition 1 can be replaced by any other norm upon, as long as the value of ε is chanced accordingly*.

**Remark 13**. *The continuous dependence of F*(**p**, **q**) *on* (**p**, **q**) (*in terms of the topologies on* $R^{\text{n}+\text{m}}$ *and L^1^; the latter space being the one where the payoff distributions live in) as implied by* Eq (3) *leads to thinking that letting ε → 0 would also cause δ → 0. This impression is not necessarily true, since the ε-condition in definition 11 can be taken as a convergence criterion when iteratively computing equilibria, while the payoff distributions can be approximated at a precision that is independent of this value ε. Indeed, the goodness of approximation is controlled by the amount of data and the parameter h chosen for the KDE or the mollifier (cf. remark 10), and as such is independent of ε. Hence, asking for a δ-deviation for the payoff distribution accounts for a nontrivial degree of freedom here*.

While existence of equilibria in mixed strategies for all finite games $A\in F^{n\times m}$ is assured (see [58]), the existence of an approximate equilibrium is not as obvious. In fact, the actual use of an approximate equilibrium is to find it within the set of regular matrix games, so that games with payoffs from F can be solved for equilibria just like a standard matrix game would be treated. Clearly, since $\mathbb{R}\;\subset \;{}^{*}\mathbb{R}$ and by virtue of the embedding *ϕ*, the set of matrix games $A\in R^{n\times m}$ forms a subclass of games of the form $A\in {{}^{*}R}^{n\times m}$, which itself covers games of the form $A\in F^{n\times m}$ by virtue of *ϕ*. A practical method to solve games over F is obtained by approximating the solution from the inner set of equilibria in matrix games with real-valued payoffs. This is the main theorem of this work, whose proof is delegated to the appendix.

**Theorem 14** (Approximation Theorem). *For every ε > 0, δ > 0 and every zero-sum matrix game* $\Gamma_{1}=A\in F^{n\times m}$ *with distribution-valued payoffs, there is another zero-sum matrix game* $\Gamma_{2}=B\in R^{n\times m}$ *so that an equilibrium in* Γ_2_ *is an (ε, δ)-approximate equilibrium in* Γ_1_.

### 7.2 Zero-Day Exploits

As a matter of consequence from the KDE approximation of empirical densities using Gaussian kernels, the approximate density in any case is supported on the entire real line. That is, the density function (5) assigns positive likelihood to the entire range (*a*, ∞), where *a* is again our risk acceptance threshold. For the specific scenario (*i*, *j*), this also means that positive likelihood is assigned to losses in the range *Z* = (max_*k*_ {*x*_*k*_} , ∞), where *x*_1_, …, *x*_*N*_ are the observations upon which our empirical loss distribution is based, and *Z* is the range of losses that were never observed. These are, by definition, exactly the events of zero-day exploits. More importantly, losses in *Z* have—by construction—a positive likelihood to occur in scenario (*i*, *j*) under the approximate RV ${\tilde{L}}_{ij}$ with density function (5).

**Fig 9:**
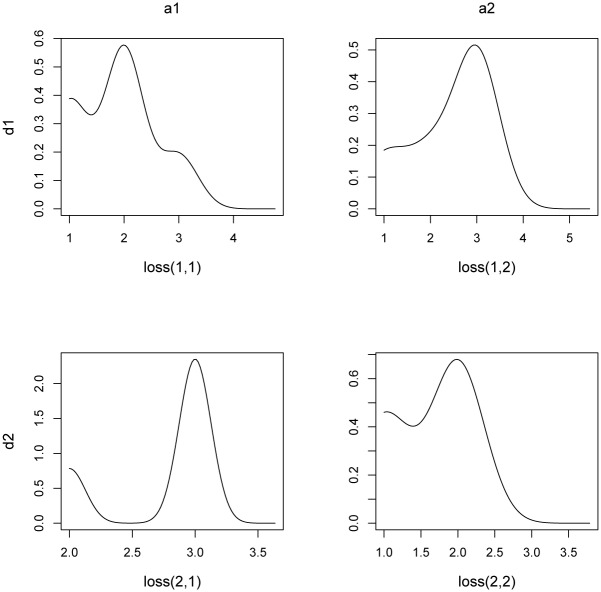
Equilibrium loss distribution for the example APT mitigation game.

In other words, no matter of whether or not we explicitly sought to model zero-day exploits, they are automatically (implicitly) taken into account by the loss density approximation technique laid out in Section 7. The specific likelihood for a zero-day exploit, as based on the information available, is simply the mass assigned to *Z* under ${\tilde{f}}_{{\hat{L}}_{ij}}$ (as defined in Eq (5)). This value increases, the more observations on higher losses are available, say, if more experts expect higher damages to occur. In that case, the kernel density estimate will put more mass on this area, thus fattening the tails of the KDE approximation Eq (5). Fig 9 will later display the equilibrium outcome of an example APT-game model, showing the “zero-day area” in gray.

In connection with the design of our games to yield optimal behavior that minimizes the chances for high losses, the likelihood for zero-day exploits is then automatically minimized, since the ⪯-optimal decisions are those that have all their masses shifted towards lowest damages as much as possible. Therefore, practically, we can adjust our modeling account for zero-day exploits by adding more pessimistic observations to the data sets from which we construct the loss distributions. But from that point onwards, the construction automatically considers extreme events in the way as we want it without further explicit considerations.

## 8 Generalizations and Special Cases

The APT modeling can be generalized to deal with multiple relevant interdependent aspects, such as different security goals (confidentiality vs. integrity vs. availability, etc.) but also taking costs into account (such as, for example, if the attack graph is enriched with information on how much an exploit would cost the attacker, or the level of skills required to mount an attack). By virtue of our embedding into the hyperreals, all the known theory from multi-criteria game theory carries over to this setting. Specifically, multi-criteria games as have been studied by [61–64] can be analyzed in exactly the analogous way in our setting. This has been done in [65], so we confine ourselves to only sketching the approach here.

### 8.1 Multiple Goals and Optimal Tradeoffs

Suppose for simplicity that only a choice between finitely many options is to be made, where risk is decreased upon increasing investments.

**Example 15** (Uninterruptible power supplies). *Imagine that a company ponders about installing additional power-supplies to cover for outages. Depending on how many such systems are available (in different subsidiaries of the company), the risk for an outage will obviously decrease. Equally clear is the increase of costs per additional system, so putting both of these outcome measures into a graph, we end up with finding the optimal tradeoff somewhere in the middle. The question is now how to find the optimum algorithmically*.

Formally and generally, let $F_{ij}^{1},F_{ij}^{2},\ldots ,F_{ij}^{d}$ be different measures of loss that all need to be accounted for in the scenario (*i*, *j*) ∈ *AS*_1_ × *AS*_2_, and let *α*_1_, *α*_2_, …, *αd* ∈ (0, 1) be weights assigned to these losses (identically for all *i*, *j*). Practically, such losses could concern (among others):

- confidentiality of information (loss distributions $F_{ij}^{1}$),
- availability of systems (loss distributions $F_{ij}^{2}$),
- security investments/costs to run defense actions (loss distributions $F_{ij}^{3}$),
- etc.

Then, [62] has shown that a vector-valued loss referring to multiple interdependent criteria can be converted into a simple (single-criteria) loss by taking

$$F_{ij}≔\alpha_{1}\cdot F_{ij}^{1}+\alpha_{2}\cdot F_{ij}^{2}+\ldots +\alpha_{d}\cdot F_{ij}^{d},$$ (11)

into the optimization. Technically, the convex combination takes us to the Pareto-front of admissible actions, and the resulting optima and Nash-equilibria are understood in terms of Pareto-optimality. In Eq (11), the addition is understood pointwise on the distribution functions, which is mathematically justified since the expectation operator is linear w.r.t. the distributions over which it is computed (hence, the moment sequences arise in the proper form). Consequently, multicriteria game theory as studied in [61–63] applies *without change* here.

The only technical constraint that applies here is that all $F_{ij}^{k}$ for all *i*, *j*, *k* must have the same support Ω⊂R. That is, we must measure the loss in a common scale, for otherwise, the above scalarization does not make sense.

#### Combining Losses of Different Nature

Different goals may be measured in individual scales (such as monetary loss being expressed as a number, loss of public reputation expressed in a nominal scale like “low/medium/high confidence” or loss of customers being an integer count). To harmonize these towards making the convex combination Eq (11) meaningful, we need to cast all these scales into a common scale *Ω* over the reals. While there is no general method to do this, practical heuristics to achieve this mainly do two steps:

1. Define an ordered set of fixed loss categories that shall apply for all goals of interest, and define the understanding of each category *individually per goal*.
2. Map all concrete (e.g., numeric) losses into the so-defined categories.

This approach is, for example, followed in many national risk management standards, such as [66–68]

**Fig 10:**
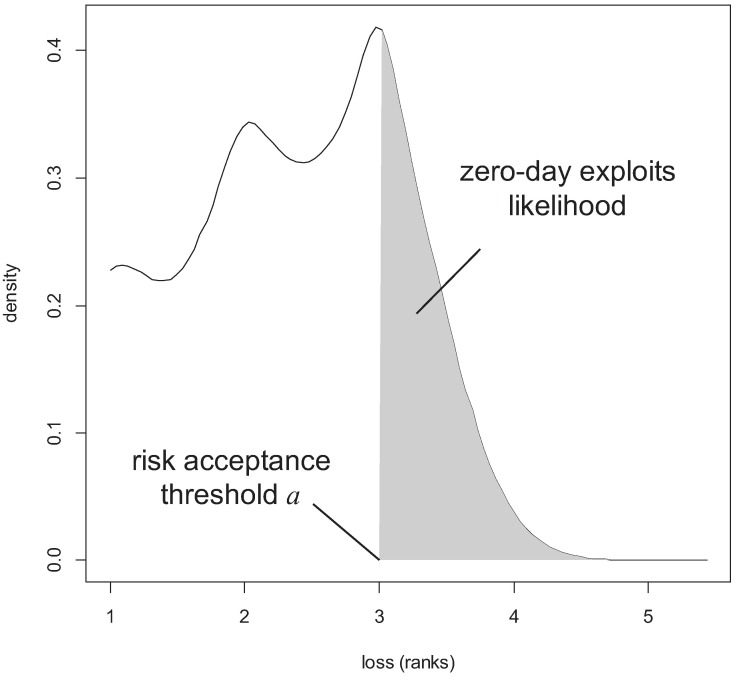
Optimal Tradeoffs (simple case).

Picking up example 15, let us assume that we seek to decide between installing between zero and five auxiliary power supply systems. Letting the priorities to be “security:costs = 60:40” (i.e., *α*_1_ = 0.6, *α*_2_ = 0.4), we find the optimal tradeoff to be between two and three additional power supplies. Finding the actual optimum is then a simple matter of comparing calculations for 2 and 3 power supplies since all other options have been eliminated; see Fig 10 (the computation on loss distributions would be identical, only taking a pointwise weighted sum of the distributions; only the resulting plot would be much less illustrative than the shown picture of real-valued functions).

### 8.2 Optimizing a (Permanent) Security Design

Since the concept of Nash equilibrium on which optimal choices are based assumes randomized (and thus repeated) actions, the question on how to apply the optimization to “permanent” countermeasures (that are not repeated actions in the classical sense) naturally appears. Indeed, this case boils down to a special case of a game in which the action space of player 2 is singleton, |*AS*_2_| = 1, and we only look at the performance of different actions, all of which may be static. Let us give two practical examples:

**Example 16** (installing anti malware systems). *Installations of anti-virus software is a standard precaution, however, given inconsistencies between reported performances and the diversity of threats, it is often advisable to install several anti malware precautions. No system ships with a guaranteed detection rate, and different systems may be differently fast in identifying and blocking threats from the outside. The decision must again be based on empirical data (reported recognition rates) and just installing the full palette of available software will not necessarily increase the protection. Thus, there is an eventual tradeoff between security and cost, for which an optimum has to be found (see Section 8.1)*.

**Example 17** (hiring security guards). *As with example 15 and as illustrated in* Fig 10, *hiring more security guards will increase likelihoods to catch an intruder, but also will increase costs. This is a case where one performance indicator is random (the chances to keep intruders outside), while the other is quite deterministic (the guard’s salaries). As before, the sought decision is a mere ⪯-optimal selection among finitely many choices*.

Generally, in both examples, the problem is to find an optimal choice among our options in *AS*_1_, while the effects of a choice are not determined by an adversarial move in the game, but rather up to our own assessment (formally, we can simulate this by defining *AS*_2_ to be singleton and contain an abstract (not further specified) action). The technical issue illustrated in both examples is the problem of ⪯-comparing *deterministic* to *random* outcomes. This is indeed easily possible and has been formally proven in [49] to work as follows: let *a* be a real value and let *B* be a real-valued RV, whose distribution is *f*_*B*_ and is supported on [1, *b*].

- If *a* ≤ *b*, then we ⪯-prefer *a* (intuitively, this is because having a constant outcome *a* is more secure to rely on than on a random effect *B*).
- If *a* > *b*, then we ⪯-prefer *B* (intuitively, since it in any case admits less damage than *a*, whose damage is guaranteed).
- If *a* = *b*, then we prefer *B* (intuitively, this is because *B* admits damages less than *a*, while the other action entails a guaranteed loss ≥ anything that can happen under *B*).

This procedure works on single criteria decisions only. If multiple criteria are to be taken into account, then we must again resort to a kernel density approximation to uniformly represent all values as RVs (thus, adding a controllable/reasonable degree of uncertainty) to the variable *a*, to be able to use the lexicographic comparisons on the convex combination of utility functions as sketched in Section 8.1.

## 9 Example Application

Continuing our running example from Section 3, much of the modeling has already been done along the vulnerability analysis described in Section 3. Indeed, the action sets *AS*_1_ and *AS*_2_ are already available in Tables 1 and 3, which makes the game a 4 × 8-matrix over yet to be specified outcomes. To simplify matters of demonstration here, let us use only two strategies out of the sets *AS*_1_, *AS*_2_, leaving the full case of our example or more extensive lists of attacks and countermeasures as an obvious matter of scaling the matrix to a larger shape. The process of finding the optimal risk mitigation strategy, however, remains unchanged between a 2 × 2- and an *n* × *m*-game, so we will illustrate the results on the smaller example without loss of generality. We stress that even though 2 × 2-games admit closed form solutions over R, the same formula in ${}^{*}R$ holds but cannot be practically evaluated in lack of an explicit ultrafilter (alas, the existence of U is assured only non-constructively). Our chosen strategies are in abbreviated form shown in Table 7.

**Table 7:** Selected Strategies for the Example.

Attack strategies	Defense actions
*a*_1_: buffer overflow exploits	*d*_1_: patching
*a*_2_: remote access exploits	*d*_2_: deactivation of services

Note that these chosen attacks are indeed generic, as buffer overflows and remote access is part of every attack listed in Table 3. In fact, this modeling appears practically reasonable, since the respective countermeasure may “break” the attack vector at any stage (as long as it breaks it at all). However, depending on how deeply the attacker has penetrated the system already, the respective countermeasures may not be effective any more. In light of this, let us follow the procedure outlined in Section 6 and assume that we have asked a group of six domain experts about their opinions on the effectiveness of countermeasures. With answers given in qualitative terms of saying that the residual *risk after mitigation* is either (H)igh, (M)edium or (L)ow, assume that the answers were as listed in Table 8 (using the abbreviations from Table 7). The concrete rating of the risk can be based on the graph-theoretic distance between the attacker and its goal (as discussed in Section 6). This view lets us convert the qualitative ratings into numeric ranks, which are *L* ↦ 1, *M* ↦ 2 and *H* ↦ 3, expressing that a “low” rating is based on the belief that the attacker has penetrated only one access control so far (e.g., gained access to machine 0), while a “high” rating means that it is already quite close to its goal (penetrated three access control systems already up to only one exploit left towards full access on machine 2; cf. Fig 2).

**Table 8:** Example Expert Assessments.

Scenario ↓ / Expert →		1	2	3	4	5	6
*a*_1_	*d*_1_	L	L	M	M	M	H
	*d*_2_		H	H		H	M
*a*_2_	*d*_1_	H		H	M	L	H
	*d*_2_	M	L	L	M	M	

Note that Table 8 shows empty cells, which correspond to cases where an expert was silent (without explicit opinion) on a specific scenario. Such missing information is only an organisational inconvenience, yet causes no technical difficulties, as in that case, we simply compile the loss distributions from the data that is available.

We define a KDE using Eq (5) for each scenario, using the data from Table 8, such as the loss distribution for the scenario (*a*_1_, *d*_2_) would, for example, be constructed from *N* = 4 data points (*x*_1_, *x*_2_, *x*_3_, *x*_4_) = (3, 3, 3, 2).

**Fig 11:**
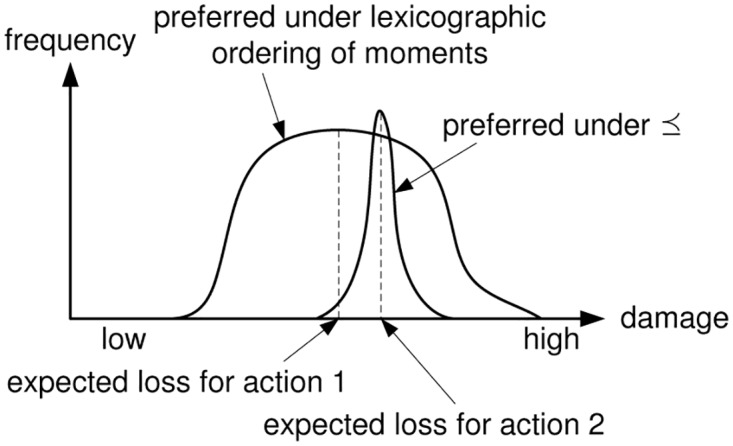
R-plot of our example APT matrix game.

We practically implemented this scheme in R [69], using the available heuristics for bandwidth selection that R provides (concretely Silverman’s rule), and obtained the distribution-valued matrix game shown in Fig 11, with the label “loss(i, j)” indicating the scenario (*d*_*i*_, *a*_*j*_) for *i*, *j* ∈ {1, 2}, with meanings as told by Table 7.

To obtain a solution, we implemented a standard fictitious play algorithm (see, e.g., [57]), with the only modification of minima and maxima being selected w.r.t. the lexicographic ordering of the derivative sequences Eq (10). The derivatives themselves are computed by evaluating Eq (9). The used risk acceptance threshold *a* ≥ 1 in our implementation defaults to the largest observation (data point) available. That is, we consider any event with consequences above “high damage” as residual zero-day exploit risk, which by construction of the ⪯-relation is minimized (cf. Theorem 7).

Taking 1000 iterations of fictitious play and rounding the result to three digits after the comma, we obtain the approximate equilibrium $\left({\tilde{p}}^{*},{\tilde{q}}^{*}\right)=\left(\left(0.875,0.125\right),\left(0.238,0.762\right)\right)$ along with the resulting equilibrium loss distribution as shown in Fig 9, and formally given as the derivative of the distribution function $F({\tilde{p}}^{*},{\tilde{q}}^{*})$ defined by Eq (3). Conceptually, this density is the same as the (well known) saddle-point value of a regular game (it plays the same role and the random loss corresponding to it satisfies the equilibrium condition w.r.t. the ⪯-relation).

Now, let us look at the practical meaning of the outcome of the game-theoretic analysis:

- The optimal way of mitigating the APT as modeled by the game in Fig 11 is to do defense action *d*_1_ with likelihood 0.875 and defense action *d*_2_ with likelihood 0.125. That is, if actions are taken daily, then we would temporarily turn down a service (or enforce a disconnection) every 8th day on average, while applying patches in the meantime, whenever they are available. Note that the randomness in the modeling *accounts* for situations where a patch may not be available at all (in that case, the event of failure of the patch occurs, and high damage may be expected. This is, however, already captured by our modeling of a random outcome, which can have different effects, including a working and failing patch at different iterations of the gameplay).
- If the system administrator adheres to the equilibrium behavior ${\tilde{p}}^{*}=\left(0.875,0.125\right)$ in choosing her/his actions, then Fig 9 is by Lemma 9 a *guarantee* concerning random damages, *irrespectively* of how the attacker actually behaves. Indeed, a (non-unique) worst-case attack behavior is delivered as the second part of the equilibrium ${\tilde{q}}^{*}=\left(0.238,0.762\right)$, which tells that approximately every fourth attack “should” be a buffer overflow, while trying remote access in the remainder of the time. If our modeling was incorrect on assuming the attacker’s behavior but correct on the possible actions (depending on how accurate the topological vulnerability scan was), then any behavior different from ${\tilde{q}}^{*}$ will give us only less chances of high damage (as follows from the definition of an equilibrium; see [18]).
- Conversely, the same also holds for the system administrator. The equilibrium condition tells that any attempt to do better by patching more often or deactivating services more or less frequently will render the loss bound $V=F({\tilde{p}}^{*},{\tilde{q}}^{*})$ void (in the sense that losses are no longer optimally distributed), and may enable stronger worst-case attack scenarios (cf. Lemma 9).

From the full equilibrium distribution that is returned by the fictitious play, we can easily compile other risk measures of interest like averages (see Eq (1)) or similar. In particular, taking the expectation of the distribution $F({\tilde{p}}^{*},{\tilde{q}}^{*})$ directly returns a quantitative risk estimate according to the common formula (1). What is more, however, is our ability to obtain additional information from the outcome, such as the variance as a measure of “stability” of the risk estimate (quantified by the fluctuation around the expected damage), or the danger of zero-day exploits, for which the area within [*a*, ∞) can be computed (shown in gray in Fig 9) as an indication and decision support. The considerable size of the gray area relative to the rest is due to the expert’s majority suspecting the risk to be high (cf. Table 8). This puts a lot of mass on the right tail of the constructed loss distributions, thus fattening the tails (induced by the KDE Eq (5)) accordingly. A less pessimistic expert assessment underlying the risk analysis would result in a smaller zero-day likelihood, respectively.

These possibilities extend much beyond the usual capabilities of (quantitative) risk management.

## 10 Discussion

### 10.1 Actions in Continuous Time

Typically, cyber warefare and APTs are not games that take rounds, but a process of actions in continuous time with no defined “stages” in the game. More precisely, we have the situation of one player likely being forced to take actions at discrete times, while the other player is free act continuously over time. For example, the security officer may be unable to become active at any (prescribed optimal) time of the day, since s/he must adhere to organizational constraints of the daily business. On the contrary, the adversary is only bound to the business organizational matters as far as it concerns the mounting of an attack. In any case, the attacker can act at any point in time (during night, during peak hours of work, etc.), while the defender must use the proper time slots to become active to minimize distortions in the actual enterprise business.

This is a remarkable qualitative difference to both, discrete and continuous time game models, covering matrix games (discrete in time) and others (e.g., [16] as a continuous time model). Our specific APT model is discrete in time (as being a matrix game), but can account for randomness in the outcome caused by a continuously acting opponent. This is a mere change to the outcome (loss) distributions to cater for an action to be interrupted, distorted or to cause more damage the longer it has happened in the past.

Theoretically, the embedding of the payoff distributions into ${}^{*}R$ equips us with the full spectrum of mathematical tools as are known and applied to construct continuous time games (this is a direction of future research from this work). Practically, we believe the application of discrete time games to more properly match the possibilities of a real-life security officer, who may take actions only at particular times, i.e., outside peak-hours of work, when devices are temporarily idle, or similar. Invoking Lemma 9 here tells us that if the defense is optimized for discrete time actions of the defender, a “continuous time attacker” may nevertheless only deviate and as such cause less damage than what the matrix game predicts.

### 10.2 On Some Modeling Issues

This section is similar to many well-known collections of “frequently asked questions”, and indeed can be taken as a guideline on how to overcome a set of usual modeling obstacles when the model shall be applied practically:

**How to deal with large attack graphs?**

As a matter of fact, attack graphs can get huge even for small infrastructures. Enumerating all strategies by finding all paths can thus become an infeasible task (as there is usually an exponential number of them). Instead, we may either simulate attacks on the nodes directly, thus defining the attack strategies not as paths in the attack graph but rather as the set of possible exploits in our infrastructure, and ask for a qualitative risk assessment based on the exploits only. Even if the infrastructure is large, having the freedom to work with qualitative assessments in our game-theoretic model eases matters of risk assessment essentially up to the same complexity as a normal risk and vulnerability assessment would require. That is, there is no conceptual obligation in our APT game model definition to work with paths in the attack graph (this is only *one* option among many), and the APT game can be defined on aggregated parts of an infrastructure, or using any other condensed or high-level view on the system.

**Where to get the losses/probabilities from?**

Probability—in general—is a notoriously opaque concept for many practitioners, and specifying probability in our model, as for any probabilistic model, is a crucial initial task. However, unlike many other techniques, our requirements are different in an important way: we do not ask an expert for a numeric estimate of a probability, but instead it suffices to ask several experts for a qualitative rating of likelihood concerning certain attacks. That is, the expert is not challenged to tell a precise number to quantify how likely an attack is, but can rather resort to saying that the success for an attack or mitigation strategy may be “low”, “medium” or “high”. This is even in alignment with recommendations of the German Federal Office for Information Security (BSI), which explicitly warns about “precise probabilities” which can misleadingly take estimates as being objectively accurate. Asking several experts about their opinions and re-normalizing the absolute frequencies into probabilities avoids the common problems of calibrating probabilistic models and naturally delivers the necessary loss distributions as we need them (see Fig 7).

Moreover, the criticism uttered against probabilities (the difficulty to specify them and the illusion of accuracy created by them) applies much more generally to many statistical models, but not as such in the model proposed here.

An explicit alternative to surveys or mere expert opinions is the use of *simulation*. Models like [28] explicitly define a continuous time simulation for a moving target defense that can be adapted. Similar models from disease spreading analysis based on percolation theory can also be used to probabilistically assess the outcome of a malware infection (say via a bring-your-own-device scenario) [53] may also directly deliver the sought loss distributions. The appeal of any such simulation is the automatism that they provide to reduce the modeling labour.

**What if a defense fails?**

Normal game theory assumes defenses to be effective in general, for otherwise, an ineffective defense could be deleted from the game on grounds of strategic dominance (cf. [70]). In admitting defense strategies to have random effects, the failure of a defense is yet merely another form of failure of an action. Thus, upon proper modeling of the chances for an action to fail, such events are naturally covered by our random loss RVs admitting multiple different outcomes, including a complete failure. For example, if patches or updates are not available with known frequencies (say, if the vendor has a “patch day”), we can assign this likelihood as the probability of high damage despite the action. Note, however, that this is *not* the freedom of choosing *not* to do the defense action at the prescribed time. Doing so would mean to deviate from the equilibrium, which results in a worse protection. Adhering to the equilibrium behavior strategy yet failing in the defense action itself is, however, covered by the allowed likelihoods for an action to fail at random.

**How to handle one-shot situations?**

The existence of optimal decisions is often based on randomized actions, which means that actions and the entire game can be repeated. Practically, we may not be able to reset the infrastructure to a defined initial condition after a damage happened. That is, the game actually changes (at least temporarily) upon past actions. Such situations are covered by the notion of dynamic (stochastic) games, which allow future instances of the game play to depend on past rounds. So, an action may be one-shot and upon failure, may create a completely new situation. Assuming that the entirety of possibilities admits a finite number of game-theoretic descriptions, we technically have a stochastic game in Shapley’s sense [56]. The usual notion of equilibrium (optimal defense) in such competitions is, however, more intricate and its existence is often tied to additional assumptions or modifications to the game (e.g., by resorting to dynamic games, or similar). The practical issue here is the concrete choice of equilibrium outcomes (discounted, averaged, etc.) to retain a practical meaning in the APT context. We avoid such difficulties here by allowing the outcome to be different in each round and determined by past iterations of the game, as long as the outcome remains *identically distributed* between rounds. If we think of the game structure itself following a stochastic process, then the loss distributions constituting the game structure may be taken as the stationary distribution of the process, under any known condition of convergence (e.g., see [71]). We will leave this as a route for future work, and close this discussion with the statement that one-shot situations are conceptually equivalent to dealing with repeatable situations, as we do not optimize the cumulative long-run average (which would assume a repeated gameplay), but rather optimally shape the distribution of the outcome for every repetition and thus also for “one-shots”.

**Is Knowledge about the Adversary’s Incentives or Intentions Required?**

Adversary modeling is often perceived necessary or at least useful in defending assets, especially in APT scenarios. However, defenses tailored to a specific guess about the adversary’s intentions or incentives may perform only suboptimal depending on the accuracy of the guess. Although a game-theoretic model can be designed to take into account adversarial payoffs if they are known, we do not actually require an accurate understanding of the adversary’s intentions or incentives.

It is important, however, to understand who the adversary is, because this is what determines its action set *AS*_2_. The more we know about the attacker, the more accurate we can model its actions, and thus reduce the possibilities for unexpected incidents. Therefore, we must stress that Lemma 9 only spares us to understand the adversary’s intentions, but *we can in no case ignore* its capabilities. Thus, we *do require an adversary model* here, yet being accurate only on the adversaries *possible actions* and on our own loss upon these.

**How to set the risk acceptance threshold *a*?**

The risk acceptance threshold *a* > 1 is here required primarily for technical reasons, i.e., to assure the boundedness of supports to ease deciding preferences among actions. Thus, as long as any such value is being defined, the theory and results remain intact. Physically, this parameter corresponds to the maximal damage that we expect to occur ever, or otherwise said, cases of damage that are covered by insurances or considered so unlikely that the risk is simply taken.

Quite obviously, the concrete choice of the threshold *a* has a substantial influence on the decisions and computed equilibria. Indeed, cutting off tails at different locations can even reverse the preference under ⪯. Therefore, this value should be chosen based on trial comparisons between actions to look for a paradoxical/counter-intuitive outcome of ⪯ (see [58] for an illustration), so that the value *a* can be set accordingly. Technically, it determines the region of loss that we would relate to zero-day exploits (as was discussed in the context of Fig 9). In any case, the particular choice of *a* is up to expertise, experience, and is seemingly out of the scope of any default procedure to choose it.

Several rule of thumbs may be defined, such as choosing *a* as a maximum quantile over all loss distributions (similar to a value at risk (VaR) approach outlined in [72]), or directly taking *a* as the worst risk assessment made or possible. Our implementation in R takes the maximum observation or most pessimistic loss assessment as the cutoff point, assuming that no more than the worst expected outcome may be expected. However, if losses are quantified in monetary terms or general business value, the lot of insurance coverage may determine the acceptable risks that can be taken.

## 11 Conclusion

Mitigating APTs on game theoretic grounds appears as a quite natural model of the competition between a defender and an attacker. The stealthiness of APT adds an element of uncertainty that original game theory covers with extended notions like stochastic games or games with incomplete information. Since these are conceptually more involved to define, we propose staying with a simpler and easier to set up model of matrix games. Deviating from classical game theory at this point, we defined a concept that allows for “direct use” of vague and uncertain information that risk management normally has to deal with. Specifically, lifting game theory from real-valued payoffs to games whose outcomes are described by entire probability distributions creates aspects of twofold interest: practically, this model equips us with the full armoury of game and decision theory to do risk management based on uncertain and even qualitative information. Theoretically, the so-generalized games come with substantially different properties than their classical counterparts, such as the non-convergence of fictitious play for a certain class of zero-sum games. The way in which these issues are tackled is not tied to applications of security and may thus be of independent interest in game theory. Finally, in using matrix games with distributions as payoffs, we tackle another aspect of APTs, which is the game being discrete time for one player but continous time for the other player. This aspect was hardly discussed in precursor work.

For security, the game theoretic perspective lets us not only compute optimal risk mitigation strategies almost directly starting from the available information, but also elegantly saves us from some matters of adversary modelling. Especially, we only need to know the attacker’s possible actions, but can work out a (multi-criteria) optimal defence in terms of our own risk scale. This is particularly useful in the context of guarding against APTs, since uncertainty is “ubiquitious” in the attacker’s capabilities, incentives, induced damages, etc. Having models that spare us the need to model all these aspects, while dealing with uncertainty in the way it comes (such as expert opinions on risk or expectations on zero-day exploits) appears as a demanding issue. Our work is intended as a first step into this direction.

## Appendix: Proof of the Approximation Theorem 14

The following arguments are partly based on [49] and close some open gaps in this preliminary draft presentation of results. The proof of Theorem 14 rests on the following lemma:

**Lemma 18**. *Let f, g ∈ C^∞^*([1, a]) *for a real value a > 1 be probability density functions. If*

$${\left({\left(-1\right)}^{k}\cdot f^{\left(k\right)}\left(a\right)\right)}_{k\in N}{<}_{lex}{\left({\left(-1\right)}^{k}\cdot g^{\left(k\right)}\left(a\right)\right)}_{k\in N},$$

*then f ⪯ g*.

*Proof*. The proof repeats the arguments in [49], and is essentially based on Lemma 5. That is, it suffices to determine which density is taking lower values in a right neighborhood of the cutoff point *a*. To this end, let us “mirror” the functions around the vertical line at *x* = *a* and look for which of *f*(*x*), *g*(*x*) grows faster when *x* becomes larger than *a*, using an induction argument on the derivative order *k*. Clearly, whichever function grows slower for *x* ≥ *a* in the mirrored view is the ⪯-preferable one by Lemma 18. Furthermore, we may assume *a* = 0 without loss of generality (as this is only a shift along the horizontal line). For *k* = 0, we have *f*(0) < *g*(0) clearly implying that *f* ⪯ *g*, since the continuity implies that the relation holds in an entire neighborhood [0, *ε*) for some *ε* > 0. Thus, the induction start is accomplished.

For the induction step, assume that *f*^(*i*)^(0) = *g*^(*i*)^(0) for all *i* < *k*, *f*^(*k*)^(0) < *g*^(*k*)^(0), and that there is some *ε* > 0 so that *f*^(*k*)^(*x*) < *g*^(*k*)^(*x*) is satisfied for all 0 ≤ *x* < *ε*. Take any such *x* and observe that

$$\begin{array}{ll} 0 & >\;\overset{0}{\underset{x}{\int }}\left(f^{\left(k\right)}\left(t\right)-g^{\left(k\right)}\left(t\right)\right)dt\;\\ & =\;f^{\left(k-1\right)}\left(x\right)-f^{\left(k-1\right)}\left(0\right)-\left[g^{\left(k-1\right)}\left(x\right)-g^{\left(k-1\right)}\left(0\right)\right]\;\\ & =\;f^{\left(k-1\right)}\left(x\right)-g^{\left(k-1\right)}\left(x\right)\;, \end{array}$$

since *f*^(*k*−1)^(0) = *g*^(*k*−1)^(0) by the induction hypothesis. Thus, *f*^(*k*−1)^(*x*) < *g*^(*k*−1)^(*x*), and we can repeat the argument until *k* = 0 to conclude that *f*(*x*) < *g*(*x*) for all *x* ∈ [0, *ε*).

For returning to the original problem, we must only revert our so-far mirrored view by considering *f*(−*x*), *g*(−*x*) in the above argument. The derivatives accordingly change into $\frac{d^{k}}{dx^{k}}f\left(-x\right)={\left(-1\right)}^{k}f^{\left(k\right)}\left(x\right)$, and the proof is complete.

*Proof of Theorem 14*. This is an actually easy matter of collecting what we have obtained in Section 7.1. First, note that w.l.o.g., we can represent the losses in **A** by distribution functions *F*_*ij*_ for *i* = 1, 2, …, *n* and *j* = 1, 2, … *m*. To ease notation in the following, let *i*, *j* be arbitrary, and abbreviate *F*_*ij*_ as *F*. We will go through a sequence of approximations of *F*, denoted as ${\tilde{F}}_{1},{\tilde{F}}_{2},{\tilde{F}}_{3}$, and ${\tilde{F}}_{4}$, respectively, and prove that the *L*^1^-approximation error of the final approximation ${\tilde{F}}_{4}$ can be made bounded by *δ* upon proper constructions of the intermediate approximations.

To get started, let us get back to the mollifier approach outlined in remark 10: we choose some *h* > 0 and define first approximation ${\tilde{F}}_{1}\left(h\right)≔F*K_{h}\in C^{\infty }$. Note that this already makes the sequence representation Eq (10) well-defined. Moreover, it is known that letting *h* → 0, the sequence ${\tilde{F}}_{1}\left(h\right)$ is *L*^1^-convergent to *F* by known approximation theorems (e.g., on page 321 in [73]). That is, we can choose a sufficiently small $h^{*}$ > 0 to have ${\tilde{F}}_{1}≔F*K_{h^{*}}$ satisfy $\|\tilde{F}\;-\;{F\|}_{L^{1}}<\delta /4$.

Note that since *K*_h*_ is supported on the entire real line, so is ${\tilde{F}}_{1}$. To recover the required bounded support, we choose some value *a* > 1 and truncate the distribution ${\tilde{F}}_{1}$ outside the interval [1, *a*]. Call the result ${\tilde{F}}_{2}$. Since ${\tilde{F}}_{1}$ is a probability distribution, it satisfies $\lim_{x\to \infty }{\tilde{F}}_{1}\left(x\right)=1$, so that we can choose *a* sufficiently large to make the truncated distribution ${\tilde{F}}_{2}$ satisfy $\|{\tilde{F}}_{1}\;-\;{{\tilde{F}}_{2}\|}_{L^{1}}<\delta /4$ again.

Since ${\tilde{F}}_{2}\in C^{\infty }\left(\left[1,a\right]\right)$ by construction and the derivatives are all continuous (and as such bounded on the compact interval [1, *a*]), we can approximate ${\tilde{F}}_{2}$ at the point *x* = *a* by a Taylor-polynomial ${\tilde{F}}_{3}=\sum_{i=0}^{K}\frac{{\tilde{F}}_{2}^{\left(i\right)}\left(a\right)}{i!}\cdot {\left(x-a\right)}^{i}$. The *i*-th derivative is analytically given by ${\tilde{F}}_{2}^{\left(i\right)}=F*K_{h}^{\left(i\right)}$ and computed numerically by virtue of Eq (9). The accuracy of the Taylor polynomial approximation is governed by choosing the order *K* of the polynomial sufficiently large. In our case, we take *K* large enough to make the approximation satisfy $\|{\tilde{F}}_{2}\;-\;{{\tilde{F}}_{3}\|}_{L^{1}}<\delta /4$.

Finally, observe that the Taylor polynomial ${\tilde{F}}_{3}$ can be represented by a finite sequence of its coefficients $\left(a_{0\;},\;a_{1},\;\ldots \;,a_{K\;}\right)\;\in {\mathbb{R}}^{K}$ with $a_{i}≔{\tilde{F}}_{2}^{\left(i\right)}\left(a\right)$. To the end of recovering a finitely truncated representation as in Eq (10), define the coefficients with alternating signs *b*_*i*_ ≔ (−1)^*i*^*a*_*i*_ for *i* = 0,…, *K*. Let us choose fixed integers *m*, *n* and round all *b*_*i*_ to *m* places before and *n* binary digits after the comma, padding with leading and trailing zeroes. Call the resulting approximate coefficients ${\tilde{b}}_{i}$, and define the respective binary number $c≔0.{\tilde{b}}_{1}∥{\tilde{b}}_{2}∥\ldots ∥{\tilde{b}}_{K}$ by simply concatenating the bitstrings representing all *b*_*i*_ in ascending order of indices and omitting the decimal points.

Observe that the number *c* is now a real value that encodes a Taylor-polynomial ${\tilde{F}}_{4}$ with approximate coefficients ${\tilde{a}}_{i}$, which differ from the coefficients *a*_*i*_ in ${\tilde{F}}_{3}$ only by a rounding error. Consequently, the maximal difference between ${\tilde{F}}_{4}$ and ${\tilde{F}}_{3}$ comes to

$$\begin{array}{ll} \underset{x\in \left[1,a\right]}{\text{max}}\left|{\tilde{F}}_{3}\left(x\right)-{\tilde{F}}_{4}\left(x\right)\right| & \leq \;\underset{x\in \left[1,a\right]}{\text{max}}\left|\overset{K}{\underset{i=0}{\sum }}\frac{\left|a_{i}-{\tilde{a}}_{i}\right|}{i!}\cdot {\left(x-a\right)}^{i}\right|\\ & \leq \;\underset{x\in \left[1,a\right]}{\text{max}}\left|\overset{\infty }{\underset{i=0}{\sum }}\frac{\varepsilon_{n}}{i!}\cdot {\left(x-a\right)}^{i}\right|\leq \varepsilon_{n}\cdot e^{a}, \end{array}$$

where *ε*_*n*_ is the maximal numeric roundoff error depending on the number *n* of digits after the comma. Since *a* is a constant, we can choose *n* sufficiently large to make $\|{\tilde{F}}_{4}\;-\;{{\tilde{F}}_{3}\|}_{L^{\infty }\left(\left[1,\;a\right]\right)}$ sufficiently small, and hence also cause $\|{\tilde{F}}_{4}\;-\;{{\tilde{F}}_{3}\|}_{L^{1}\left(\left[1,\;a\right]\right)}<\delta /4$ ultimately (this is a consequence of using Hölder’s inequality to show that convergence in the function space *L*^*p*^ implies convergence in *L*^*q*^ for *q* < *p* if the underlying support is compact; see page 233 in [74]).

Collecting the approximations obtained along, we end up finding that $\|F\;-{{\tilde{F}}_{4}\|}_{L^{1}}\;\leq \;\|F\;-{{\tilde{F}}_{1}\|}_{L^{1}}\;+\;\|{\tilde{F}}_{1}\;-{{\tilde{F}}_{2}\|}_{L^{1}}\;+\;\|{\tilde{F}}_{2}\;-{{\tilde{F}}_{3}\|}_{L^{1}}\;+\;\|{\tilde{F}}_{3}\;-{{\tilde{F}}_{4}\|}_{L^{1}}\leq 4\cdot \frac{\delta }{4}=\delta$, as required.

Indeed, repeating these steps for every entry in the matrix $A=\left(F_{ij}\right)\in F^{n\times m}$, we end up with a matrix of respective values $B=\left(c_{ij}\right)\in R^{ij}$ representing finite sequence approximations of *F*_*ij*_. Moreover, observe that the construction of the matrix **B** is such that the numeric order between two entries is exactly the lexicographic order on the sequence of rounded coefficients (this is due to the concatenation and the fact that all numbers *b*_*i*_ are represented use the same number of digits before and after the comma). Hence, Lemma 18 tells that the order of choices made in the game **B** equals the ⪯-order of choices that would be made in the game **A**. Consequently, an equilibrium in **B** is also an equilibrium in **A** since ≤ on the so-obtained *c*-values in **B** equals ⪯ on the original loss distributions in **A**.

Since **B** is a regular matrix game, we can invoke fictitious play (or linear optimization) to compute an approximate (or even accurate) equilibrium (${\text{p}}^{*}$, ${\text{q}}^{*}$) in **B** at any desired precision *ε*. By construction of **B** and Eq (3), an equilibrium (${\text{p}}^{*}$, ${\text{q}}^{*}$) will approximate the equilibrium payoff in **A** (whose existence is assured by Nash’s theorem holding in the hyperreal space ${}^{*}R$ by the transfer principle [42]). This completes the proof.

As a final remark, note that the representation of the Taylor-polynomial within the real number *c* is compatible with the multi-criteria optimization as outlined in Section 8.1. To see this, observe that the convex combination Eq (11) is a linear operation whose result remains within the same bound as the inputs. Thus, there will be no “overflow carry” from one coefficient to the next in the representation *c* of the Taylor-polynomial.
