# Correction: Defending Against Advanced Persistent Threats Using Game-Theory

**DOI:** 10.1371/journal.pone.0317848

**Published:** 2025-01-16

**Authors:** Stefan Rass, Sandra König, Stefan Schauer

In Related Work subsection of Introduction, there is an error in the first sentence of the fifth paragraph. The correct sentence is: Whereas AECID (and similar tools) are detective measures (as they trigger alerts based on specific events that have happened already), our approach in the following is preventive in the sense of estimating and minimizing the risk of a successful APT from the beginning (cf. Section 1.2).

In the Organization of the paper subsection of Introduction, there is an error in the fourth sentence. The correct sentence is: Section 4 introduces the theory of how decisions can be made if their outcome is rated through an entire probability distribution object (rather than a number), and Section 5 takes this basis to define games, equilibria and to highlight similarities but also an important qualitative difference between the so-generalized games and their classical counterparts (among others, (Bürgin et al., 2021) showed that fictitious play can converge to a point that is not necessarily an equilibrium, which led to the introduction of a lexicographic Nash equilibrium as a new concept in (Rass et al., 2021); we will go into more details later in Section 5).

The references are: Bürgin V, Epperlein J, Wirth F. Remarks on the tail order on moment sequences. arXiv:210410572 [math]. 2021.

Rass S, König S, Schauer S, Bürgin V, Epperlein J, Wirth F. On Game Theory Using Stochastic Tail Orders. arXiv:210800680 [math]. 2021

There are sequence errors in Table 3. Please see the correct Table 3 here.

**Table 3:** APT scenarios (adversary’s action set AS2, based on Fig 2).

1	execute(0) → ftp rhosts(0,1) → rsh(0,1) → ftp rhosts(1,2) → rsh(1,2) → local bof(2) → full access(2)
2	execute(0) → ftp rhosts(0,1) → rsh(0,1) → rsh(1,2) → local bof(2) → full access(2)
3	execute(0) → ftp rhosts(0,2) → rsh(0,2) → local bof(2) → full access(2)
4	execute(0) → rsh(0,1) → ftp rhosts(1,2) → rsh(1,2) → local bof(2) → full access(2)
5	execute(0) → rsh(0,1) → rsh(1,2) → local bof(2) → full access(2)
6	execute(0) → rsh(0,2) → local bof(2) → full access(2)
7	execute(0) → sshd bof(0,1) → ftp rhosts(1,2) → rsh(1,2) → local bof(2) → full access(2)
8	execute(0) → sshd bof(0,1) → rsh(1,2) → local bof(2) → full access(2)

In Definition 2 of the Modeling Uncertainty for Decision-Support section, there is an error in the second sentence of the third bullet. The correct sentence is: In the latter case, we assume the density *f*_*L*_ to be continuous and piecewise polynomial over a finite partition of its support.

In the Modeling Uncertainty for Decision-Support, the last paragraph is missing. The last paragraph should be: We stress that the conditions of Def. 2 only mildly constrain the set of choices, since (by Weierstraß’ theorem), all smooth distributions allow a polynomial approximation in the desired sense, if they are compactly supported

In Definition 6 of the Modeling Uncertainty for Decision-Support section, the last paragraph is incomplete. The correct paragraph is: That is, the action with the higher likelihood of extreme damage is less favorable, and upon a tie (equal chances of large damages), the likelihood for the next smaller risk level tips the scale, etc. Rephrasing the classical saddle-point condition in terms of such a lexicographic order leads to a new concept that coincides with (standard) Nash equilibria only in the 1-dimensional case (as observed by [31]). In higher dimensions, corresponding to non-scalar losses, such as categorical loss distributions, an optimum w.r.t. the lexicographic order, does not necessarily also induce a saddle point in the sense of *≼*. We will hence use different names to distinguish classical from lexicographic equilibria, later in Section 5 and onwards.

In Practical Meaning of ⪯-Preferences subsection of Modeling Uncertainty for Decision-Support, there is an error in the second paragraph. The correct paragraph is: This is just an intuitive re-statement of Lemma 5. However, and remarkably, the converse to it is also true, if the density is piecewise polynomial (see [31,32] as an extension to the original Thm. 2.14 in [46]):.

In the Games and Equilibria subsection of Practical Decision Making, there is an error in the first paragraph. The correct paragraph is: With the uncertain outcome in a scenario of defense *i* vs. attack *j* being captured by a (perhaps empirical) probability distribution *L*_*ij*_, and the complete set of distributions being totally ordered w.r.t. ⪯, it is a simple and straightforward manner to define matrix games and equilibria in the well-known way, but will need to bear in mind that the resulting concepts will not exactly resemble (classical) equilibria in all senses, as we will explain later. For convenience of the reader, we give the necessary concepts and definitions here from classical game theory.

In the Games and Equilibria subsection of Practical Decision Making, there is an error in seventh sentence of the third paragraph. The correct sentence is: This is the essential technical process of our game-theoretic APT risk mitigation strategies, whose optimality is that of a lexicographic Nash equilibrium [32], which is, in the 1-dimensional case (only), the same as a standard Nash equilibrium (see [31] for a detailed example, and [48] for a formal treatment of the classical case). In general, a lexicographic equilibrium respects goal priorities, which are here equal to the ordering on the loss scale, taking highest losses as most important to avoid (and breaking ties by moving to the next lower loss category). Similar as a standard equilibrium, a lexicographic equilibrium penalizes unilateral deviations, but in doing so, opens up a possibility for the second player to improve its own revenue in a (less important) other goal (e.g., causing more likely damage of a lower loss category). The theoretical facts about (real-valued) Nash equilibria in games, by the transfer principle, translate likewise to hyperreal terms. The practical difference relates to computability, since the defenses that we can find (algorithmically) in games over loss distributions are obtained from lexicographic equilibria. We will disambiguate the two hereafter by speaking about (standard) Nash equilibria to mean the classical concept, and lexicographic (Nash) equilibria to denote the other.

In the Games and Equilibria subsection of Practical Decision Making, there is an error in first sentence of the fourth paragraph. The correct sentence is: As for standard games, it can be shown that the saddle-point value $V\left(A\right)=\underset{p\in S\left(AS_{1}\right)}{\text{max}}\underset{q\in S\left(AS_{2}\right)}{\text{min}}F(p,q)$ is invariant w.r.t. different (standard) Nash equilibria, and that equilibria defined w.r.t. ≼ exist (and can be generalized to standard Nash-equilibria in *n*-person games in the canonic way).

In the Practical Decision Making section, there is a sentence missing between second and third sentences of the sixth paragraph. The sentence missing is: However, the result is still not just a Nash equilibrium over the hyperreal numbers, as was observed by [31], but has a lexicographic optimality property that is nonetheless appropriate for our matters of risk management.

In the APTs as Games section, there is an error in the third sentence of the ninth paragraph. The correct sentence is: On path 4, the distance to full_access(2) is 3 nodes (ftp_rhosts(1, 2) → rsh(1, 2) → local_bof(2)), while on path 5, the distance is only 2 nodes (rsh(1, 2) → local_bof(2)).

In Lemma 9, there is an error in the third sentence of its description under APTs as Games section. The correct sentence is: If the saddle-point values of the zero-sum matrix game **A** is V (**A**), and V (**A**, **B** is any standard equilibrium payoff in the bimatrix game induced by A, B, then we have:

$$V\left(A,B\right)≼V(A)$$

provided that the defender plays a zero-sum equilibrium strategy (induced by **A**) in both games, the zero-sum game ***A*** and the bi-matrix game *(****A***, ***B****)*.

In the Practical Computation of Optimal Defenses, there is an error in the third paragraph. The correct paragraph is: Lemma 9 directly follows from the definition of standard Nash equilibria, and is a well known fact; cf. [18] for a more elaborate discussion. The computation of equilibria in the sense of Lemma 9 requires hyperreal arithmetic, but lexicographic Nash equilibria are computable by conventional means only, and the bound in (4) then remains valid w.r.t. a descending order of categories on the loss scale. This is nothing but a risk-averse optimization of worst-case outcomes.

In the Practical Computation of Optimal Defenses, there is an error in the second and third sentences of the fourth paragraph. The correct sentences are: In particular, observe that the upper bound Eq (4) is independent of the adversary’s payoff/incentive structure B, and optimizes the chances to suffer worst-case losses (and breaking ties by optimizing the losses in descending order of categories). The irrelevance of B in the upper bound tells that we do not require any information about the adversary’s intentions or incentives, as V (A) can be determined only based on the defender’s possible losses, and for a lexicographic order of losses, corresponding to a worst-case avoiding defense.

In the Practical Computation of Optimal Defenses, there is an error in the second sentence of the fifth paragraph. The correct sentence is: Fortunately, however, these issues do not apply for our matrix games here, since lexicographic Nash equilibria can still be computed using fictitious play (FP) [47, 48, 57] which uses practically doable ⪯-comparisons only.

In the Practical Computation of Optimal Defenses, there is an error in second sentence of the tenth paragraph. The correct sentence is: However, depending on how “deep” the stack is made, we can reach a lexicographic equilibrium at arbitrary precision. This is made rigorous in the following definition:

In Definition 11 under Practical Computation of Optimal Defenses, there is an error in the second sentence of the description. The correct description is: We call a strategy profile $\left({\tilde{p}}^{*},{\tilde{q}}^{*}\right)\in R^{n+m}$ an (*ε*, *δ*)-approximate equilibrium, if there is an equilibrium $\left(p^{*},q^{*}\right)\in R^{n+m}$ (in the zero-sum game **A**) such that both of the following conditions hold:

1. ${\left\|\left({\tilde{p}}^{*},{\tilde{q}}^{*}\right)-\left(p^{*},q^{*}\right)\right\|}_{\infty }<\varepsilon$, and
2. ${\left\|{\tilde{F}}^{*}-F^{*}\right\|}_{L^{1}}<\delta$,

where the equilibrium payoffs ${\tilde{F}}^{*}$ and F* are defined by Eq (3) upon the equilibrium and its approximation.

In Theorem 14 under Practical Computation of Optimal Defenses, there is an error in its description. The correct description is: For every *ε* > 0, *δ* > 0 and every zero-sum matrix game $\Gamma_{1}=A\in F^{n\times m}$ with distribution-valued payoffs, there is another zero-sum matrix game $\Gamma_{2}=B\in R^{n\times m}$ so that an equilibrium in Γ_2_ is an (*ε*, *δ*)-approximate lexicographic equilibrium in Γ_1_.

In Zero-Day Exploits subsection of Practical Computation of Optimal Defenses, there is an error in the fourth sentence of the second paragraph. The correct sentence is: Fig 9 will later display the lexicographic equilibrium outcome of an example APT-game model, showing the “zero-day area” in gray.

In Example Application section, the seventh paragraph is incorrect. The correct paragraph is: Taking 1000 iterations of fictitious play and rounding the result to three digits after the comma, we obtain the approximate lexicographic equilibrium $\left({\tilde{p}}^{*},{\tilde{q}}^{*}\right)=((0.875,0.125),(0.238,0.762))$ along with the resulting equilibrium loss distribution as shown in Fig 9, and formally given as the derivative of the distribution function $F\left({\tilde{p}}^{*},{\tilde{q}}^{*}\right)$ defined by Eq (3). Conceptually, this density is the same as the (well known) saddle-point value of a regular game (it plays the same role and the random loss corresponding to it satisfies the equilibrium condition w.r.t. the ⪯-relation) more precisely, the lexicographic order on the probability masses distributed over the loss range.

In Example Application section, there is an error in the first sentence of the second bullet in the eighth paragraph. The correct sentence is: If the system administrator adheres to the (lexicographic) equilibrium behavior ${\tilde{p}}^{*}=(0.875,0.125)$in choosing her/his actions, then Fig 9 is by Lemma 9 a guarantee concerning random damages, irrespectively of how the attacker actually behaves.

In Example Application section, there is an error in the first sentence of the ninth paragraph. The correct sentence is: From the optimal distribution that is returned by the fictitious play, we can easily compile other risk measures of interest like averages (see Eq (1)) or similar.

In the Appendix: Proof of the Approximation Theorem 14, there is an error in the first sentence of the third paragraph. The correct sentence is: For the induction step, assume that *f*^(*i*)^(0) = *g*^(*i*)^(0) for all *i* < *k*, *f*^(*k*)^(0) < *g*^(*k*)^(0), and that there is some *ε* > 0 so that *f*^(*k*)^(*x*) < *g*^(*k*)^(*x*) is satisfied for all 0 < *x* < *ε*.

In the Appendix: Proof of the Approximation Theorem 14, there is an error in the third sentence of the third paragraph. The correct sentence is: Thus, *f*^(*k*−1)^(*x*) < *g*^(*k*−1)^(*x*), and we can repeat the argument until *k* = 0 to conclude that *f*(*x*) < *g*(*x*) for all *x* ∈ (0, *ε*).

In the Appendix: Proof of the Approximation Theorem 14, there is an error in the fourth sentence of the 12^th^ paragraph. The correct sentence is: Consequently, an equilibrium in **B** is a lexicographic equilibrium in **A** since ≤ on the so-obtained *c*-values in **B** equals ⪯ on the original loss distributions in **A**.

In the Appendix: Proof of the Approximation Theorem 14, there is an error in the second sentence of the 13^th^ paragraph. The correct sentence is: By construction of **B** and Eq (3), an equilibrium (p*, q*) will approximate a lexicographic equilibrium payoff in **A.**
